# Overcoming Depression on the Internet (ODIN): A Randomized Controlled Trial of an Internet Depression Skills Intervention Program

**DOI:** 10.2196/jmir.4.3.e14

**Published:** 2002-12-17

**Authors:** Greg Clarke, Ed Reid, Donna Eubanks, Elizabeth O'Connor, Lynn L DeBar, Chris Kelleher, Frances Lynch, Sonia Nunley

**Affiliations:** ^1^Kaiser Permanente Center for Health ResearchPortland ORUSA

**Keywords:** Internet, depression, cognitive therapy, self care, randomized controlled trial

## Abstract

**Background:**

Psychoeducational programs are increasingly being delivered over the Internet. We created an Internet-based, cognitive therapy, self-help program to be used as a stand-alone intervention for mild-to-moderate depression, or as an adjunct to traditional services for more severe depression.

**Objective:**

To evaluate the effectiveness of a web-based intervention program to reduce depression in a randomized, controlled trial

**Methods:**

In a private, nonprofit health maintenance organization, we mailed recruitment brochures to two populations: depressed adults (n = 6994) who received traditional medical services for depression, and an age/gender matched sample of nondepressed adults (n = 6996). Participants consenting to the study were randomized to either the experimental Web site (n = 144), or a no-access control group (n = 155). Participants in both groups were free to obtain nonexperimental, usual care services for their depression. All participants completed an on-line version of the Center for Epidemiological Studies Depression Scale (CES-D) at enrollment and at 4-, 8-, 16- and 32-weeks after enrollment. Mean intake scores were in the severely depressed range. 74% of participants completed at least one follow-up assessment. Unfortunately, most intervention participants accessed the Internet site infrequently.

**Results:**

We failed to find an effect for the Internet program across the entire sample. However, post-hoc, exploratory analyses revealed a modest effect among persons reporting low levels of depression at intake.

**Conclusions:**

The negative results might have resulted from infrequent patient use of the Internet site, or a more seriously depressed sample than the intervention was intended to help. Future studies should focus on recruiting persons with mild to moderate levels of depression, and on increasing participant use of the Internet site.

## Introduction

Many persons with clinical depression or subdiagnostic symptoms of depression obtain traditional medication and/or psychotherapy services [1,2]. However,many of them, as many as 50% or more, do not obtain such traditional treatments [3]. The reasons for this seeming disconnect between disorder and treatment are only partially understood, but include barriers to care such as inadequate insurance coverage and limits on treatment [4].

Contributing to this pattern of undertreatment, some patients may elect not to seek professional help, at least initially. Persons who do not seek traditional treatments for depression may use less-intensive, self-help materials, such as books, pamphlets, videotapes, or computer programs, collectively called *bibliotherapy* [5]. For persons with subdiagnostic symptoms of depression, bibliotherapy may be an appropriate, severity-matched, and less-intensive first alternative to traditional services. For persons with more severe depression, it may be a valuable augmentation to traditional services.

Bibliotherapy materials for depression [6,7] usually employ a well-researchedapproach, such as cognitive-behavioral therapy (CBT), which has been shown to be effective when delivered face-to-face. Typically, the most novel aspect of bibliotherapy is the medium in which it is presented. Patients follow a standardized treatment in book form and work through it more or less independently. Contacts with therapists, if any, typically support or facilitate the primary bibliotherapy.

Research suggests that bibliotherapy is effective for depression [8-10]. In fact, a recent meta-analysis of bibliotherapy for emotional/behavioral problems, including depression, found that bibliotherapy was as effective as therapist-administered treatments [5], although it is possible that this finding is true only for less-severe depression.

### Beyond Bibliotherapy

Depression self-help books and videotapes, while valuable, have several drawbacks. Health providers may find it difficult to accurately monitor a given patient's progress and use of bibliotherapy materials. In addition, these materials are static; that is, they are not tailored to an individual patient's particular problems. Books or pamphlets also presume a minimum reading level for use. Another limitation of printed material is the inability to employ recorded sound or video vignettes. These multimedia features may make self-help more accessible to members with lower reading skills, may engage all patients to a greater degree, and may prove more effective at conveying self-help skills [5].

We have recently developed an Internet-based, depression self-help program that addresses many of these drawbacks [11]. We call this site *unattended*, in that live personnel do not staff it. Instead, it provides self-guided, interactive tutorials to help users acquire antidepression skills such as cognitive restructuring [12]. In this respect, it is more like an interactive booklet than an analog of live therapy. This unattended aspect of the program distinguishes it from Internet sites where mental health professionals conduct person-to-person psychotherapy or counseling via e-mail exchanges with patients.

To the best of our knowledge, this program was unique and was not duplicated by any other depression resource on the Internet at the time of this study. While many other mental health and depression Internet sites provided information (such as the Depression Awareness, Recognition and Treatment site maintained by the National Institute of Mental Health, http://www.nimh.nih.gov/publicat/depressionmenu.cfm),all of those that we found were limited to explanations of causes and treatments. None of these informational sites offered training in behavior change skills. Research from related behavioral fields such as drug prevention suggest that information-only interventions are inadequate to bring about positive change, and that skills-training interventions are needed to achieve detectable and enduring improvement [13,14]. Our experimental Internet program offered both information and direct training in self-help, cognitive restructuring skills.

In contrast to the informational Internet sites, Marks [15] summarizes computerized programs that provide treatments for depression and other mental health problems. Several of these programs have interactive and multimedia features in common with this Internet program. However, none of these programs were available on the Internet at the time of this study, which means they were not available to a whole community in the same manner as an Internet site.

Another related but distinct category of studies has reported successful results obtained with Internet-based, mental health interventions targeting problems such as eating disorder risk factors [16,17], weight loss [18], and panic disorder [19]. These studies have in common the use of the Internet to deliver at least some components of the experimental intervention. However, at least a portion of each of these interventions includes person-based counseling or psychotherapy offered either face-to-face or through the Internet (similar in many ways to therapy delivered via telephone). In contrast, the current study presents the results of a randomized trial of an Internet depression intervention that relies exclusively on a psychoeducational tutorial that does not employ any monitoring or live interaction between participants and clinical staff. To the best of our knowledge, at the time of this study there were no other unattended but interactive interventions for depression on the Web. Since that time, there has been a report of a similar self-guided depression skills training program on the Internet [20], but to date it has not been evaluated in a randomized, controlled trial.

This study is also distinct from other randomized trials of Internet health programs because of its effectiveness design [21,22]. That is, we evaluated the Internet program in the context of a variety of other *usual care* health services, which were provided to the participants outside the research protocol. The comparison condition was usual care in a health maintenance organization (HMO), rather than an enforced no-treatment comparison condition.

We hypothesized that, compared to persons randomized to a *no-access* control group, persons who were randomized to have access to the Internet depression site would demonstrate greater reduction in self-reported depression symptoms.

## Methods

### Subjects and Recruitment

The sampling frame was approximately 430000 members enrolled in the Kaiser Permanente Northwest (KPNW) health maintenance organization, in northwest Oregon and southwest Washington. The study research center is located within the HMO, and is scientifically autonomous, self-governed, and committed to conducting public-domain research. The Human Subjects Committee for the HMO approved all study procedures.

**Figure 1:**
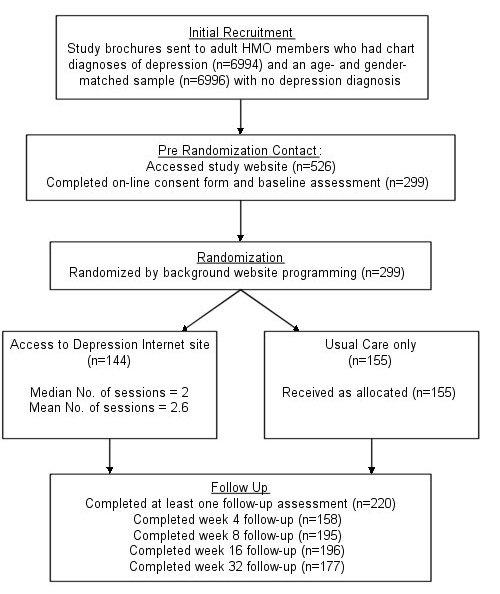
Study flowchart

The study recruitment pool was generated in several cohorts over a 7-month period in 1999. We employed the HMO's electronic medical record (EMR) systems to identify 2 groups of potential participants (see Figure 1). The first group was adults (n = 6994 over 7 cohorts) who received traditional HMO medical services (medication, psychotherapy) in the previous 30 days in association with a recorded diagnosis of depression in the EMR. We called this first group *depressed* cases, although we do not have any independent research confirmation of their clinical mood diagnosis such as with a structured diagnostic interview. The second group was *nondepressed* adults (n = 6996 over 7 cohorts) who did not receive any depression-related HMO services and did not have an EMR diagnosis of depression, but who were age- and gender-matched to the first group.

All potential participants were mailed an identical study recruitment brochure, which contained the following statements:

We are inviting you to participate in a new Internet-based program for people who may be feeling sad or depressed. We are evaluating whether this new service is helpful. We are mailing this brochure to thousands of HMO members. We do not know which members will benefit from this new Internet service, so we are mailing this letter to many people, only some of whom may be feeling depressed.

The brochure explained more details of the study, and provided the research Internet address. No further contacts were made; it was left to the initiative of invited individuals to take the first step and visit the study Internet site.

At the study home page (the starting point for the study and the intervention), invited members were asked to enter their HMO health plan number, birth day, and birth month. If these did not match the stored data for invited members, the individual was not permitted to proceed. Of a total of 13990 brochures mailed, a total of 526 participants entered confirmed health plan numbers at the study home page. This represented an initial engagement rate of 3.8% of all invited members. However, because not all invited members had Internet access, it is useful to estimate the engagement rate among those with the ability to participate. An internal survey conducted by the HMO health plan at the end of 1999 found that approximately 62.6% (n = 745/1190) of HMO members in this region had Internet access either at home or work or both. Therefore, these 526 participants may also be viewed as an initial engagement rate of 6.0% out of 8758 invited HMO members (62.6% of 13990) estimated to have Internet access.

The 526 participants entering the study site were asked to read the on-line consent form, and to indicate their consent by selecting an *I agree to take part* button. Participants were next asked to complete the on-line assessment battery (described below). Following this, 299 members (56.8%) agreed to be randomized to conditions. Randomization was accomplished immediately by a random-assignment algorithm encoded into the Web site programming: 144 participants were granted access to the experimental Internet site (the intervention condition), and 155 were not granted access (the usual care control condition). Seventy-six members of the nondepressed recruitment group (n = 6996) were enrolled in the study (1.1% of those invited) and 223 of the depressed recruitment group (n = 6994) were enrolled in the study (3.2% of those invited). Compared to HMO members who were mailed a brochure but who were not randomized, those in the randomized sample were more likely to be female (76% of the randomized sample vs 70% of the nonrandomized sample, *P*= .03) but did not differ in age.

Following the on-line consent and data collection (see below), subjects randomized to the Internet depression program were immediately linked to the home page for the intervention program. Subjects randomized to the control condition were immediately linked to the Kaiser Permanente Online home page, a Web site maintained by the health plan for HMO members only [23]. At the Kaiser Permanente Online site, participants could obtain noninteractive information about health concerns, including depression. They could also send a question to an HMO advice nurse or pharmacist, or request an appointment at local medical centers. In both conditions, subjects were free to continue or initiate any other traditional health care services for depression or other psychiatric problems, and/or access Kaiser Permanente Online or any other Internet health resource.

### Assessment Battery

While enrolling in the study, participants were asked to provide information on their age, gender, marital status, ethnicity, and educational attainment. Table 1 presents these characteristics by experimental condition. Participants completed an on-line version of the Center for Epidemiological Studies Depression Scale (CES-D) [24] a self-report measure of the frequency of 20 depressive symptoms over the past week using a 5-point Likert scale.

Reviews of computerized depression assessment methods support their reliability, validity, and equivalence to paper versions, with patients generally more truthful in their answers and often preferring computerized methods for assessing sensitive areas such as suicide and depression [25]. For example, computerized and paper-and-pencil versions of the CES-D correlate at a very high level [26].

Subjects in both conditions were sent e-mail reminders to return to the study Web site at 4-, 8-, 16-, and 32-weeks post-randomization, to complete on-line CES-D follow-up questionnaires. If subjects failed to respond to two e-mail reminders for any single follow-up point, study staff attempted to reach them by telephone. Participants were sent a $5 e-mail gift certificate redeemable with an Internet merchant (Amazon.com) for each completed assessment, for a possible total of $25.

Follow-up completion rates were 53% (n = 158) at 4-weeks post-randomization, 65% (n = 195) at 8-weeks, 66% (n = 196) at 16-weeks, and 59% (n = 177) at 32-weeks post-randomization. Overall, 220 participants (74%) completed at least one follow-up assessment. Compared to participants who completed at least one follow-up assessment (baseline CES-D mean = 30.0; SD = 12.2), subjects who completed only the baseline assessment had slightly higher baseline CES-D scores (mean = 33.3, SD = 11.2; unpaired t297 = 2.10, 2-tailed *P*< .05) but did not differ with respect to experimental condition, age, gender, or recruitment group.

### Intervention

**Figure 2:**
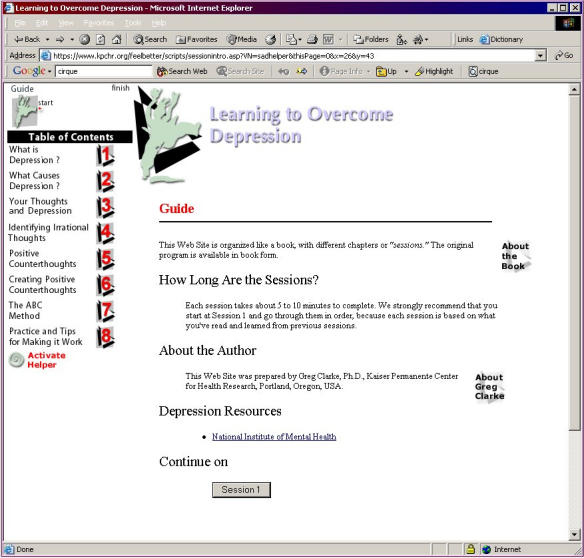
Screenshot of the ODIN program (for further screenshots see multimedia appendix)

The Internet intervention was a self-paced, skills training program focusing on the acquisition and use of cognitive restructuring techniques [12,27]. A non-research version of this program, identical to the research program but without the consent and assessment, was made available for public viewing after the trial [11]. Figure 2 shows a screenshot of the program; further screenshots are available in the multimedia appendix at the end of this paper.

Much of the Internet site content was adapted from group CBT psychotherapy manuals [28,29] that have been successfully employed in several face-to-face randomized trials [30-34]. Theskills training program was organized much like a brief booklet, with 7 *chapters*. Each chapter presented a new skill or component technique, and offered interactive examples and practice opportunities.

A representative section of the program is the *Thought Helper*, made available to participants after they had been taught the basics of identifying negative thoughts and generating positive counter-thoughts. Participants typed their negative or irrational thought into a text box, then clicked on a *search* button. The Web-server computer searched a predefined list of 200 negative thoughts for ones that best matched the negative thought submitted by the participant, and returned a screen with the most likely matches. Participants selected the returned negative thought that they felt was closest to their original. The program then returned a list of 2 to 5 possible positive counter-thoughts relevant to that belief. The participant was then encouraged to create a personalized positive counter-thought, using relevant portions of the provided examples as they saw fit, and enter it into the Web site for storage.

Other interactive aspects of the Web site included an automatically-scored CES-D questionnaire embedded into the first and last sessions (as well as the follow-up assessments); feedback on improvement across time; and cartoons that taught the basics of cognitive restructuring, with participants clicking on cartoon character dialog balloons to identify, for example, irrational beliefs and positive counter-thoughts.

We elected not to incorporate live video or audio in this initial version of the program because we wanted to make this program available to all participants including those with slow connections to the Internet. However, we are preparing newer versions of this program (to be tested in subsequent randomized trials) which have live video vignettes and exercises, to take advantage of the increasing availability of high-speed, high-bandwidth Internet connections, such as cable modems.

Participants randomized to the intervention arm of the study were able to sign-in and use the Web site whenever they wanted, for the entire duration of the study. The modal number of sessions was 1 (41.0% of users), the median was 2 sessions, the mean was 2.6 (SD = 3.5), and the range was 1 to 20 sessions. Overall, this suggested a low level of use of the depression skills training site.

### Health Care Utilization

Computerized data systems provided encounter data for HMO inpatient and outpatient services, prescriptions, lab tests, emergency room visits, etc. For this trial, participant use of non-HMO health care services was not assessed.

### Analytical Plan

All analyses were intent-to-treat. We examined continuous CES-D scores using random effect regression analyses, modeling an unstructured covariance matrix, with slope and intercept as random effects [35,36]. We estimated both linear and quadratic effects for our data, as these models best fit the data. The linear trend indicates the direction and rate of change, while the quadratic trend indicates whether the rate of change increased or decreased at some point during the observation period. However, only the significance for linear trends is reported, as this effect is relevant for hypothesis testing.

### Sample Size

Determining the needed sample for this trial was difficult, given the lack of prior Internet randomized trials in mental health. However, extrapolating from reviews of the relevant mental health bibliotherapy literature [5], we identified an upper limit effect size (ES) of .55 for the current study. However, all participants in this trial were also free to obtain traditional usual care depression treatments, which might result in fewer differences between study conditions. Therefore, we selected a more conservative ES of .30 to .35 as more appropriate for this trial. For a sample calculation, we assumed a 2-sided alpha = 0.05, power = .80, and a standard deviation of 8.5 for the CES-D (the main dependent variable), derived from the depressed adult sample originally used to validate this scale [37]. This resulted in a needed sample of between 256 and 350 for the specified ES range (.30-.35). We selected the midpoint sample size of 300 as our goal.

## Results

### Comparability of Conditions

The experimental and control groups did not differ with respect to recruitment group, gender, age, or baseline CES-D score (Table 1).

**Table 1:** Comparison of experimental conditions on baseline demographics

	Intervention (n = 144)		Control (n = 155)		Significance
	Mean	SD	Mean	SD	
Age	43.3	12.2	44.4	12.4	.44
% Female	73.6%	*---*	77.4%	*---*	.44
% Minority	5.8%	*---*	5.8%	*---*	.99
% Married	60.3%	*---*	64.0%	*---*	.51
% College graduate	45.4%	*---*	39.4%	*---*	.29
% Depressed at case-finding	74.3%	*---*	74.8%	*---*	.92

### Depression

We found no differences between the control and experimental conditions on self-reported depression (CES-D) over the study period, indicating a lack of a treatment effect. Table 2 presents the depression scale scores across assessment points for the total sample (uppermost rows).

**Figure 3:**
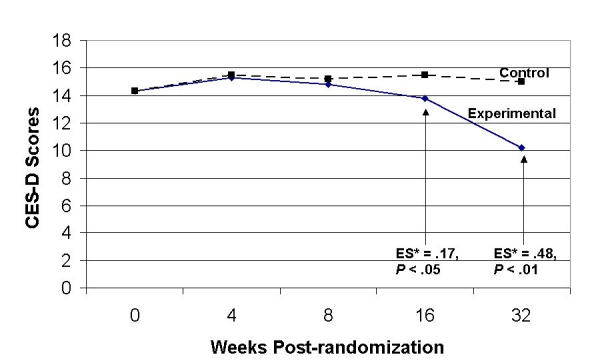
Self-reported depression outcomes for subjects reporting low baseline depression (CES-D scores < 20)

Table 2 also presents depression scale results for several subgroups. These analyses were exploratory, and thus were not powered to detect effects within the relatively small subsamples. Subgroups included (a) subjects recruited from among depression cases (n = 223); (b) subjects recruited from among nondepressed controls (n = 76); (c) subjects with high baseline depression scores (CES-D < 20) (n = 236); (d) subjects with low baseline depression scores (CES-D < 20) (n = 63); (e) female subjects (n = 226); (f) male subjects (n = 73); (g) subjects age 45 or older (n = 144); and (h) subjects younger than age 45 (n = 155). There were no significant intervention effects across the entire study duration for any of these subgroups. However, among subjects who reported lower baseline depression scores (Figure 3), experimental participants were significantly less depressed than the relevant control subjects at the 16-week follow-up (Effect Size [ES] = .17, *P*< .05) and 32 week follow-up points (ES = .48, *P*< .01).

### Dose-adjusted Effects

*Dose* analyses were conducted for the participants randomized to the Web site intervention. We predicted CES-D scores from the total number of sign-ins to the Web site (our measure of dose). The random effects regression analyses did not show a significant effect for dose (F = 0.95, *P*= .33 for sign-in-by-time effect).

### Health Care Services

In the 12 months following randomization, we found no differences in the use of health care services or psychoactive medications, including mental health outpatient visits (55.1% of treatment cases vs 48.3% of usual care cases), nonmental health outpatient visits (94.2% vs 92.9%), tricyclic antidepressant dispensings (16.3% vs 10.5%), Selective Serotonin Reuptake Inhibitor (SSRI) — antidepressant medications — dispensings (47.1% vs 49.0%), bupropion dispensings (20.0% vs 15.9%), lithium carbonate dispensings (5.6% vs 3.2%), all other antidepressant dispensings (20.6% vs 17.7%), benzodiazepine dispensings (20.4% vs 24.1%), and dispensings of any of above medications (79.1% vs 78.5%). We did find a significant difference in venlafaxine dispensings (2.8% for treatment cases vs 8.5%; chi-square1 = 4.4, *P*< .05), but given the number of comparisons this is probably not meaningful.

**Table 2:** Self-reported depression outcomes (CES-D) for total samples and subsamples

	Baseline		4 Week		8 Week		16 Week		32 Week		Significance
	Mean	SD	Mean	SD	Mean	SD	Mean	SD	Mean	SD	
**Total sample**											.86
Intervention (n = 144)	30.5	12.3	23.1	11.9	22.4	11.4	21.7	13.3	21.3	13.1	
Control (n = 155)	31.2	11.7	26.2	13.3	22.4	13.5	22.7	12.6	23.0	14.0	
**Depression cases**											.12*
Intervention (n = 107)	30.7	12.9	23.1	11.9	23.7	11.9	23.0	13.5	22.2	12.8	
Control (n = 116)	31.3	11.5	26.7	13.1	23.7	14.0	23.2	12.8	25.5	14.2	
**Nondepressed cases**											
Intervention (n = 37)	30.0	10.6	23.2	12.3	18.6	8.7	17.8	12.3	18.6	13.9	
Control (n = 39)	30.7	12.4	24.6	14.1	19.1	11.9	21.0	12.0	16.0	10.8	
**High baseline CES-D cases**											.12†
Intervention (n = 112)	35.1	9.5	26.3	11.6	25.1	11.0	24.8	13.6	25.6	12.5	
Control (n = 124)	35.4	8.7	29.3	12.4	24.6	13.0	24.5	12.4	25.2	13.6	
**Low baseline CES-D cases**											
Intervention (n = 32)	14.3	5.2	15.3	8.9	14.8	8.7	13.8	8.8	10.2	6.6	
Control (n = 31)	14.3	5.1	15.25	10.4	15.2	13.0	15.5	11.2	15.0	12.4	
**Female**											.85*
Intervention (n = 106)	31.5	12.2	23.2	12.5	22.5	11.3	21.6	12.9	21.6	13.6	
Control (n = 120)	31.4	11.9	25.9	14.0	22.6	13.6	23.2	12.2	22.5	13.6	
**Male**											
Intervention (n = 38)	27.6	12.3	22.8	10.4	22.2	11.7	22.0	14.8	20.6	11.8	
Control (n = 35)	30.4	11.2	27.2	10.8	21.8	13.4	20.7	14.1	24.7	15.3	
**Age 45 or older**											.95†
Intervention (n = 69)	28.6	11.8	22.4	10.5	21.7	9.3	21.0	12.3	21.9	11.9	
Control (n = 75)	28.2	11.4	23.4	13.2	20.5	14.8	21.6	14.2	21.5	14.6	
**Younger than age 45**											
Intervention (n = 75)	32.3	12.6	23.7	13.0	23.1	13.0	22.3	14.3	20.7	14.3	
Control (n = 80)	34.0	11.3	28.8	13.0	24.4	11.9	23.7	11.1	24.3	13.4	

*P* value for the interaction term of sex x treatment group x time (test of whether the effect of treatment on CES-D score change differed by sex)

*P* value for the interaction term of age group x treatment group x time (test of whether the effect of treatment on CES-D score change differed by age group)

## Discussion

This intervention improves on currently-available traditional bibliotherapy in several respects such as multimedia capabilities, automated scoring of questionnaires, and remote accessibility. Despite these advantages, or perhaps even because of these differences, we were unable to detect a main intervention effect for the Internet program. We conducted a series of exploratory analyses among subgroups of participants, in an attempt to better understand why we were unable to find positive effects in the overall sample. However, we also failed to find positive intervention effects among most of the subgroups, although there was a small benefit for those participants who entered the study with lower levels of depression. Because of this possible subgroup benefit, we believe this intervention requires careful examination in further studies.

There are several possible reasons for this overall lack of effect. Because they are newly developed, the Internet intervention materials themselves may have content shortcomings. That is, curative elements that are present in the hard copy bibliotherapy books and pamphlets [6] may be missing or inappropriately delivered in this Internet program. Despite these specific issues, the medium itself should improve the accessibility of bibliotherapy for a subgroup of patients likely to benefit from guided self-care. We are examining the intervention materials and improving the content for 2 subsequent trials (see below).

A related issue is whether an unattended, psychoeducational approach to delivering behavioral or lifestyle interventions via the Internet is less potent than conducting person-directed counseling through the same media. The previously conducted studies of Internet-based interventions for eating disorders [16,17], weight loss [18], and panic disorder [19] employed counselors or therapists who composed e-mail or bulletin board (chat) responses to participant questions and problems. It may be that the general absence of benefit seen in this study is due to the lack of the caring, supportive therapeutic alliance that is an integral part of in vivo psychotherapy. However, this doesn't explain how traditional bibliotherapy materials such as books achieve significant effects [5], since these materials also lack the personal relationship that is characteristic of live therapy.

Another likely reason for the lack of intervention main effects is that the recruitment procedures attracted a more seriously depressed sample than intended, resulting in participants who may have been too depressed to have benefited from such a low intensity, self-help program. Some evidence supports this explanation. Radloff [37] suggests a "serious depression" cutoff score of 16 or greater on the CES-D, the depression scale employed in this study. She also notes that only 5% of adults in a representative community sample have CES-D scores 28 or greater. This suggests that our sample, with a mean baseline CES-D of approximately 31, would be among the most depressed persons in a normal population. Further evidence comes from the exploratory analyses for persons with low baseline CES-D scores; experimental participants were significantly less depressed than the relevant control subjects at the 16- and 32-week follow-up points. However, given the large number of exploratory analyses in various subgroups, these findings must be viewed as tentative and hypothesis generating. We are preparing a subsequent randomized trial to test this finding in a more systematic manner, by specifically recruiting a much larger sample of participants with low-grade, subdiagnostic depression.

Yet another explanation for the lack of main effects assumes that both the sample and the intervention content were appropriate but that participants did not return to use the Internet site frequently enough to obtain full benefit. This would be analogous to persons who attend only one or two psychotherapy sessions. However, the dose analyses failed to demonstrate that outcome was associated with frequency of use of the Internet site, even when baseline depression severity was controlled. Nonetheless, to address this issue we have just completed enrolling 259 participants in yet another randomized trial similar to this study, but employing several low-intensity methods (e-mail messages, live telephone calls, postcards) to remind subjects randomized to the Internet program to return to the site, complete more of the content, and potentially obtain more benefit.

This study was limited in several ways that may have contributed to the absence of effects. Most importantly, attrition at follow-up was quite high, with follow-up rates at any one point ranging from 53% to 66% (although three quarters of the participants completed at least one follow-up assessment). While the lost participants were similar to retained participants on almost all baseline characteristics (except for of slightly lower depression scores), this high attrition rate reduces our confidence in the observed results. Nonetheless, given that we relied primarily on e-mail assessment reminders and the assessment was conducted via an Internet-administered questionnaire, these retention/attrition rates may represent what can be expected of this new medium where the norm is *surfing*(rapidly jumping from one Internet site to another).

Another major limitation of this study was our reliance on a single, self-report measure of depression. In our previous depression randomized trials [30] we have often employed research psychiatric interviews to ascertain diagnoses. However, this methodology is not amenable to the Internet medium at present. We could have conducted research interviews by telephone or in-person, but did not because of cost and effort limitations in this preliminary study. In addition, we were somewhat concerned that the nonspecific, semi-therapeutic impact of several hours of research diagnostic interviewing over the life of the study would potentially swamp the small therapeutic benefit expected from the Internet intervention. This is not an altogether speculative concern, but has been considered as a possible reason for null findings in another, much larger randomized trial in the alcoholism field [38].

All these issues remind us that we are at the very threshold of this burgeoning field, and that we know very little about the circumstances and processes that will optimize the delivery and acceptance of these interventions. We are in an unusual situation relative to the development and testing of in vivo psychotherapies. By the time controlled research studies were mounted to test traditional, person-to-person psychotherapy, therapists had been delivering these services in non-research settings for decades and knew a great deal about the process and parameters of service delivery. In contrast, studies of the use of the Internet to deliver mental health interventions are being conducted before widespread, non-research service delivery has occurred via this medium. Therefore, there is no accumulated clinical lore about how to best provide Internet services; we are blazing this trail as we progress. This study, and the others planned and underway, will help address some of these issues.

## Abbreviations

CBT: Cognitive Behavioral Therapy
CES-D: Center for Epidemiological Studies DepressionScale
EMR: Electronic Medical Record
ES: Effect Size
HMO: Health Maintenance Organization
KPNW: Kaiser Permanente Northwest
ODIN: Overcoming Depression on the Internet
SSRI: Selective Serotonin Reuptake Inhibitor

## Multimedia Appendix

Screenshots of the ODIN intervention; including interactive skills training, and some representative cartoons.

Note that the "usual care" (control) KPOnline site is not shown because the most relevant screen shots include bulletin board entries by actual HMO members asking clinical questions to pharmacists and psychiatrists. The Human Subjects Protection committee of the authors' institution has ruled that these screens cannot be shown.
