# Supplementary material for: Overcoming Depression on the Internet (ODIN): A Randomized Controlled Trial of an Internet Depression Skills Intervention Program
Source: J Med Internet Res. 2002 Dec 17;4(3):e14. doi: 10.2196/jmir.4.3.e14 (PMC1761939; doi:10.2196/jmir.4.3.e14)

## Slide 1
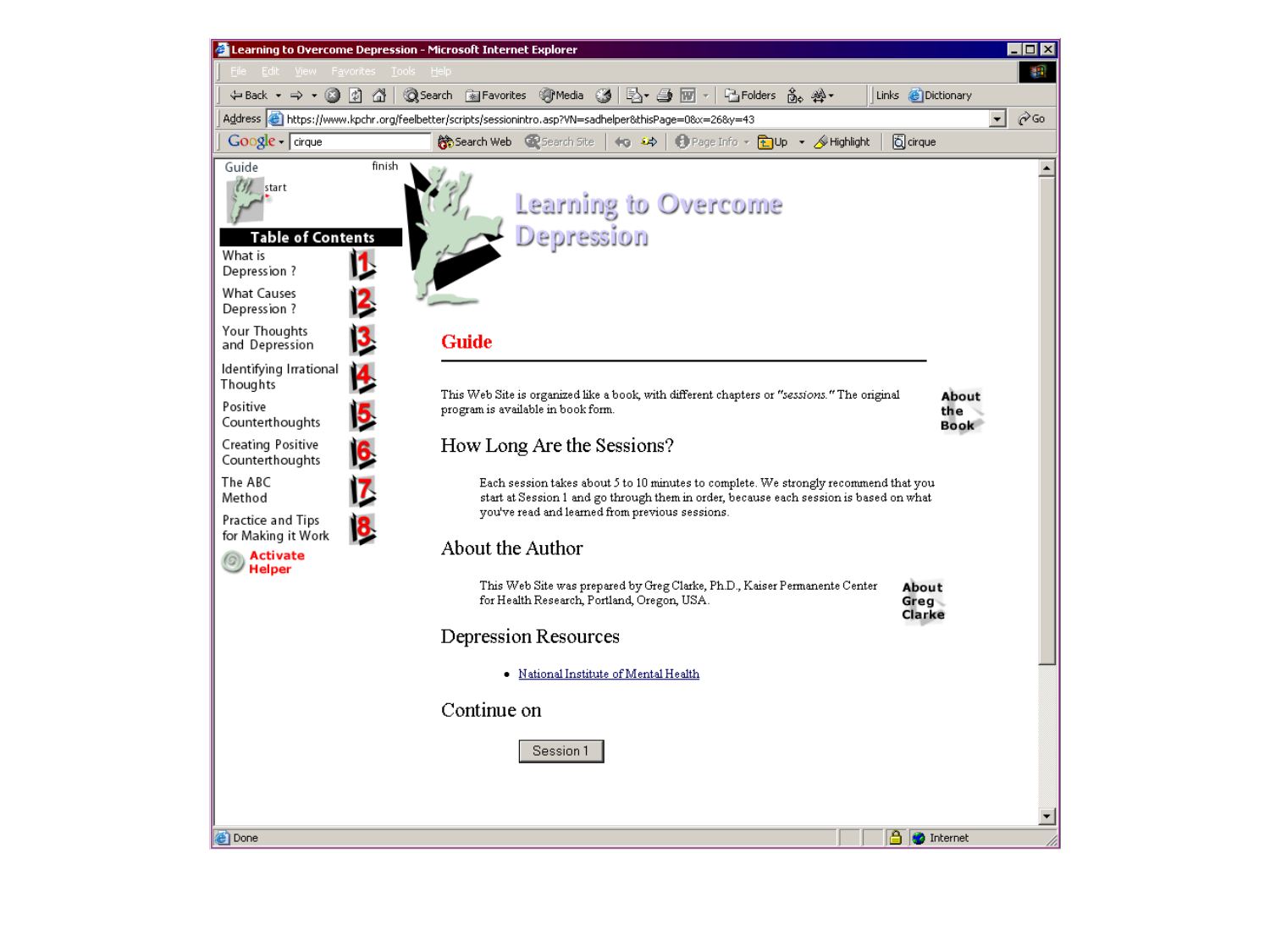

## Slide 2
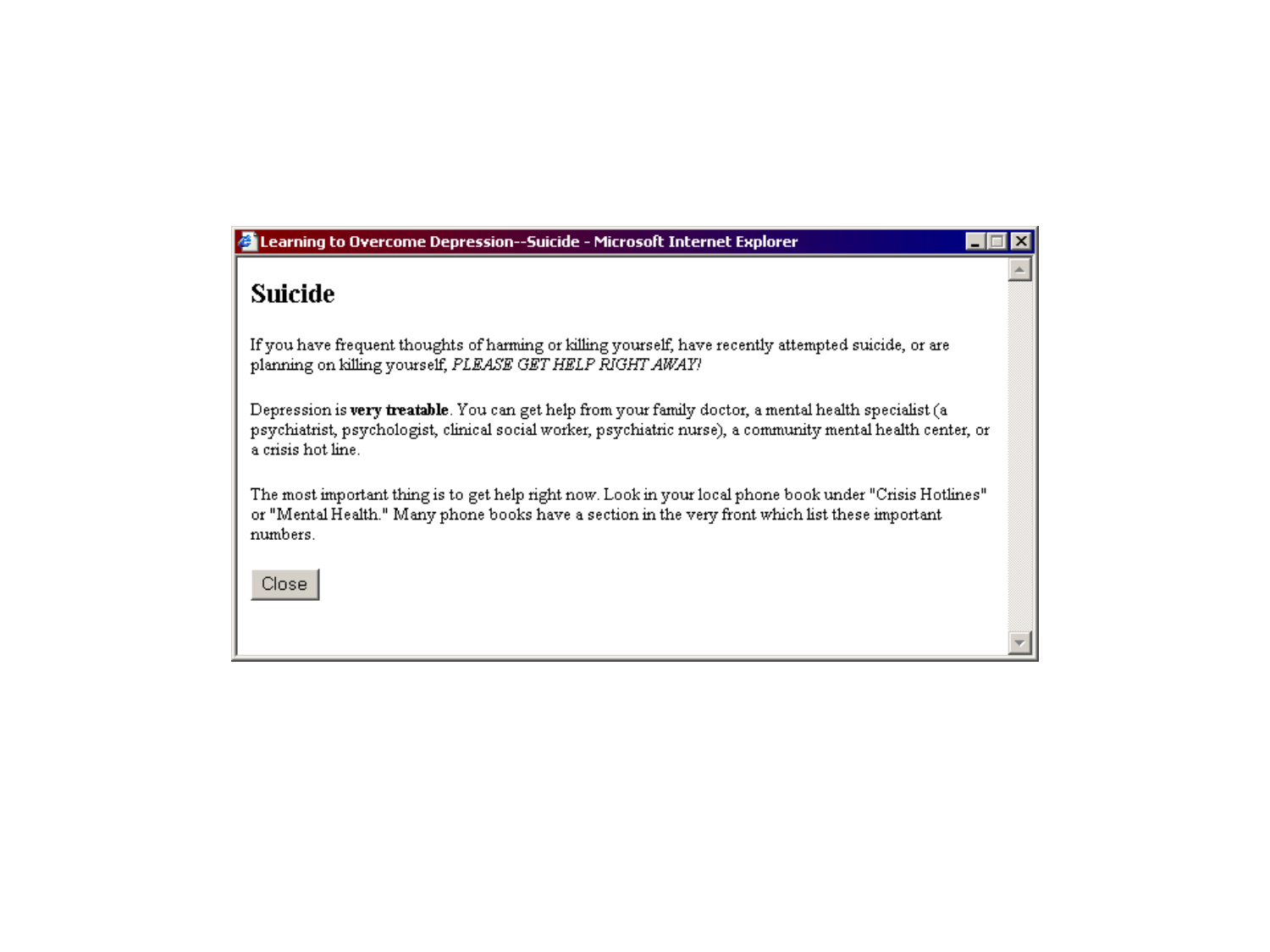

## Slide 3
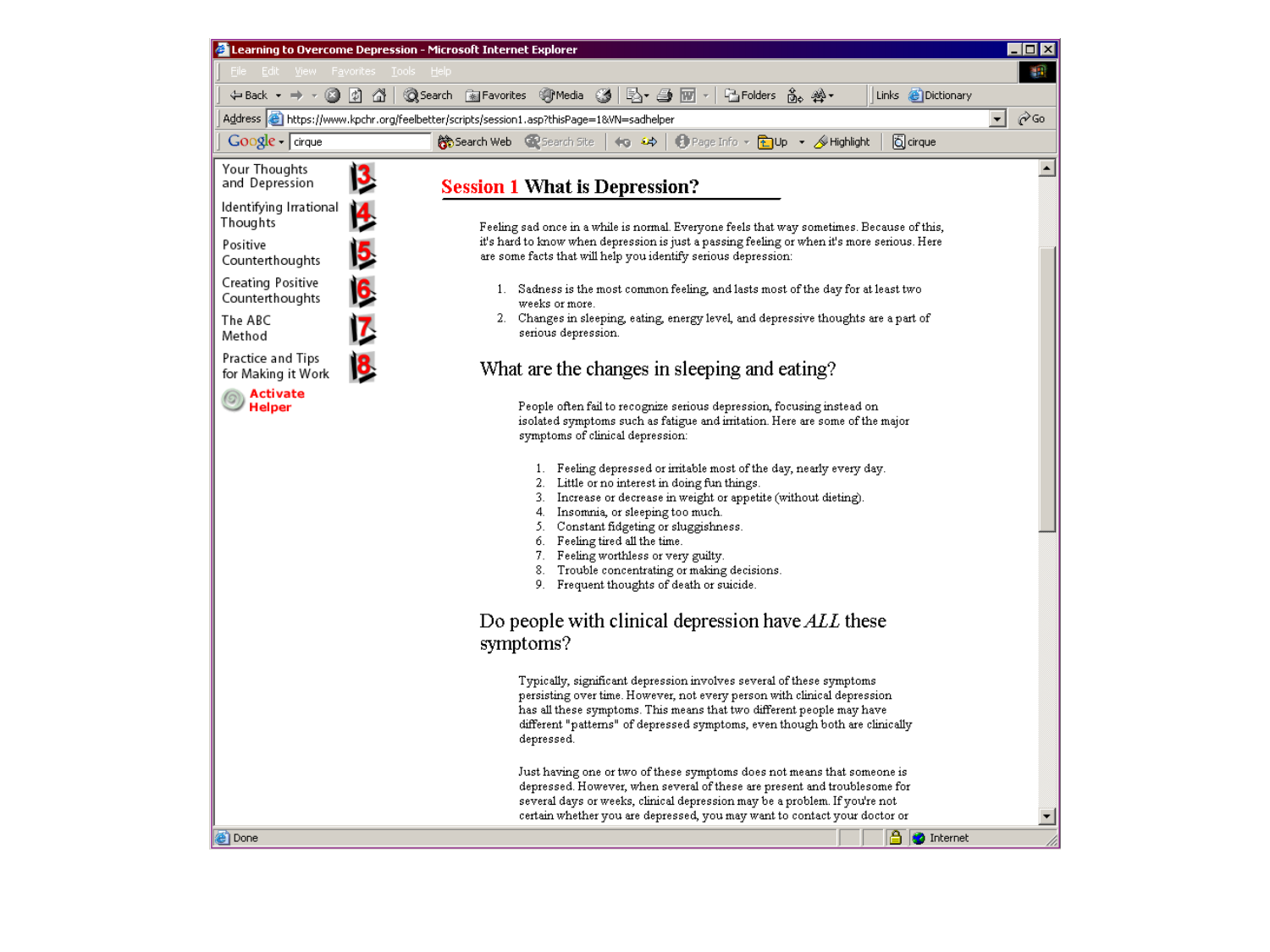

## Slide 4
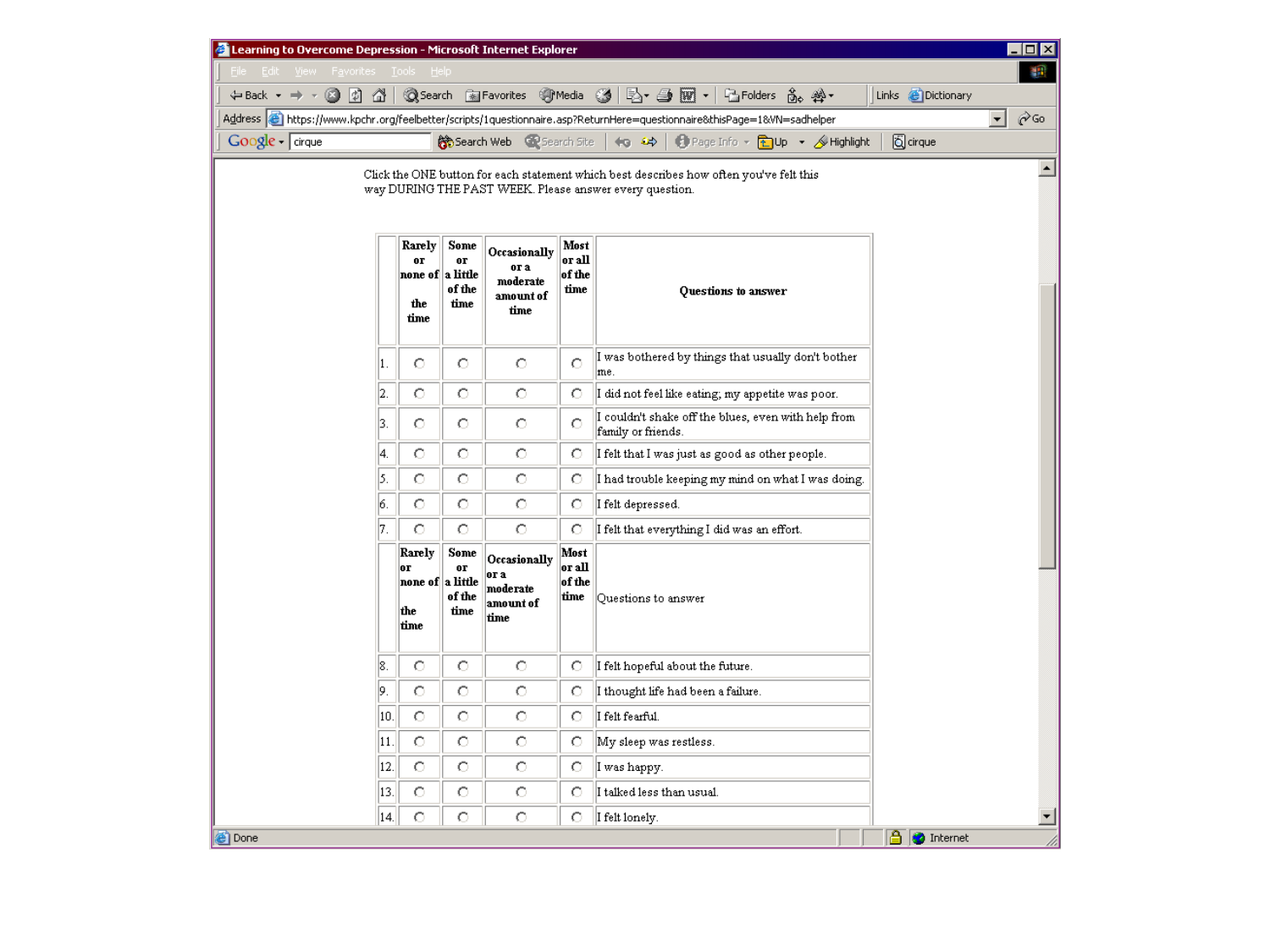

## Slide 5
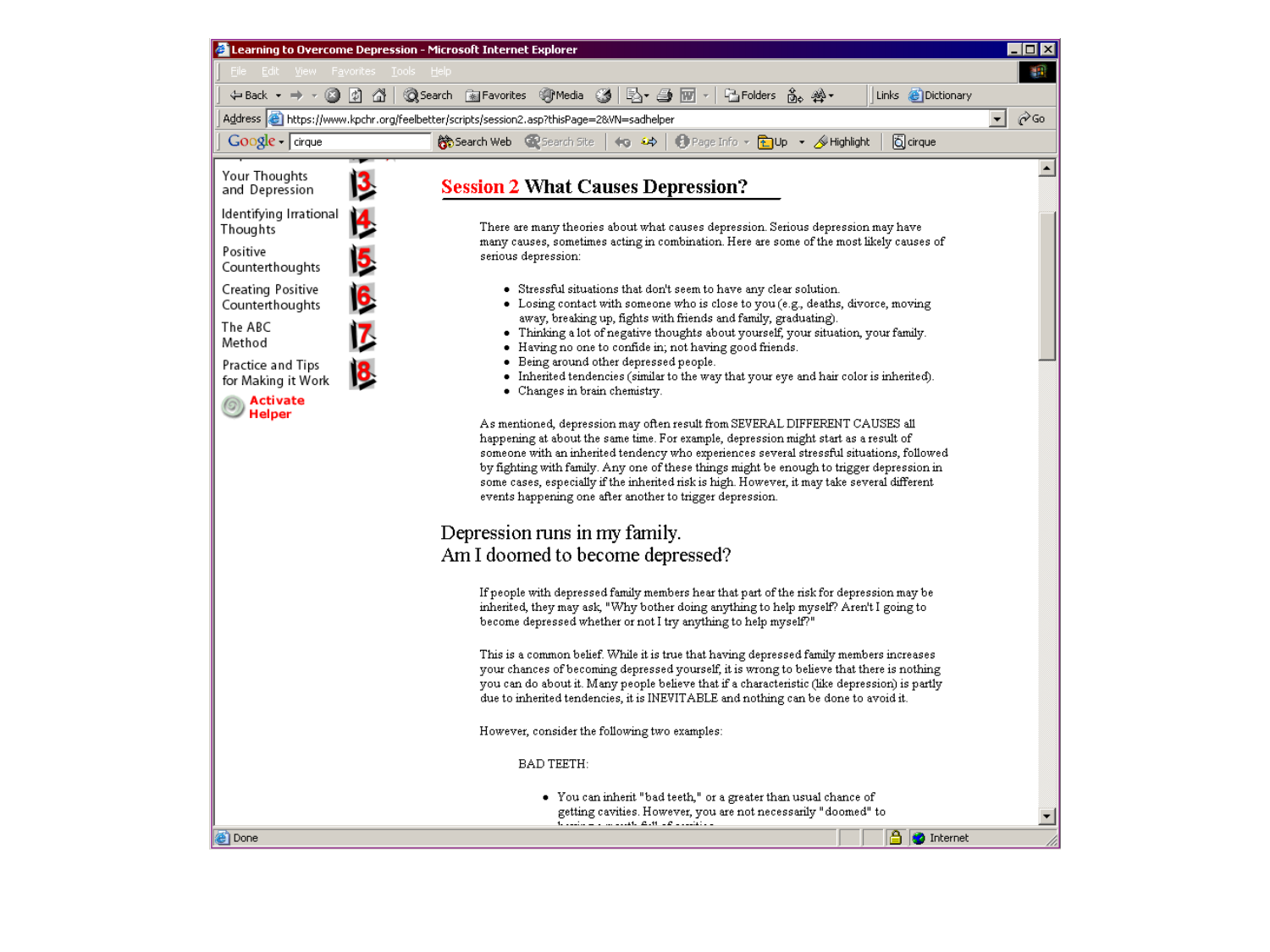

## Slide 6
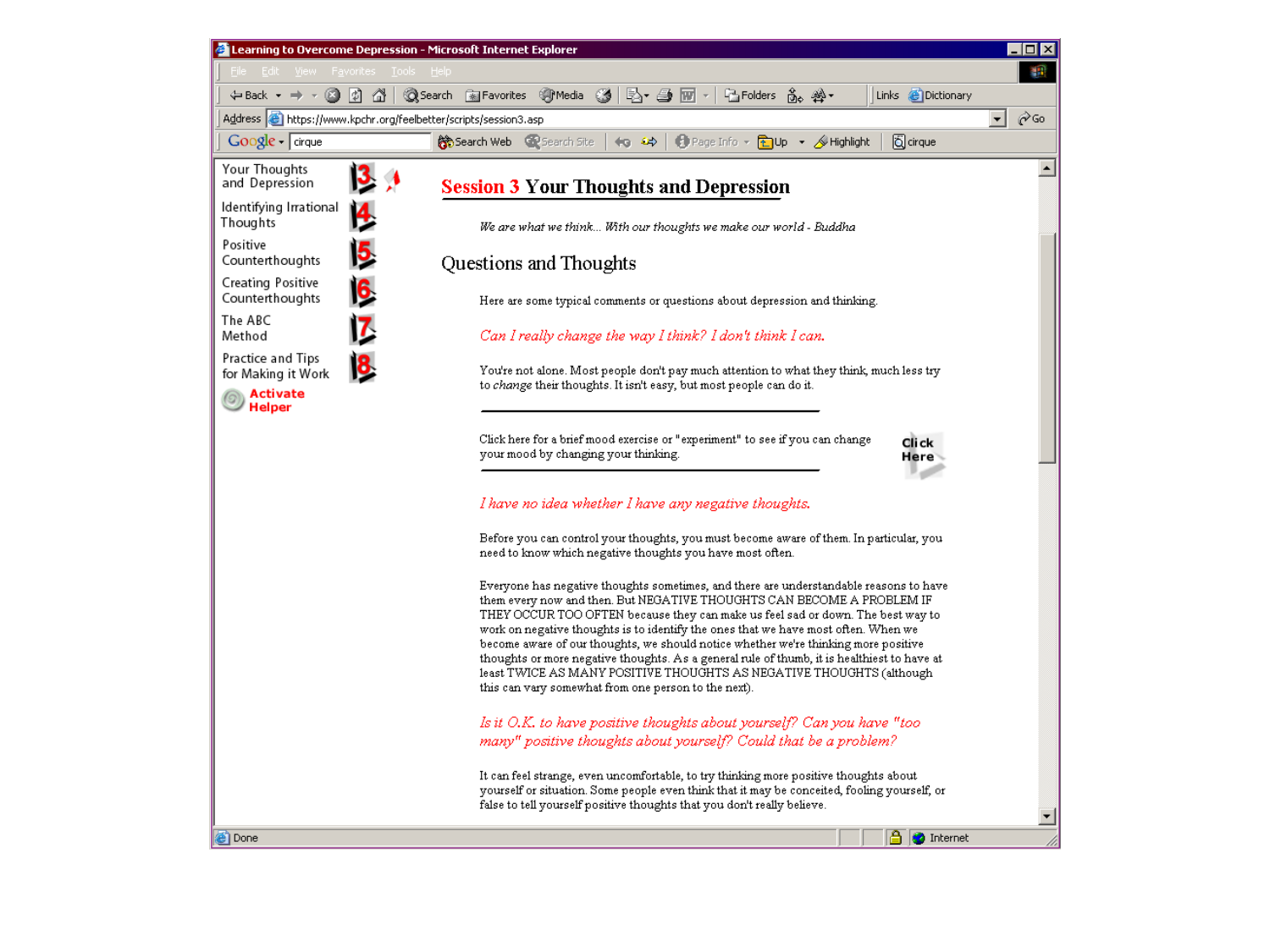

## Slide 7
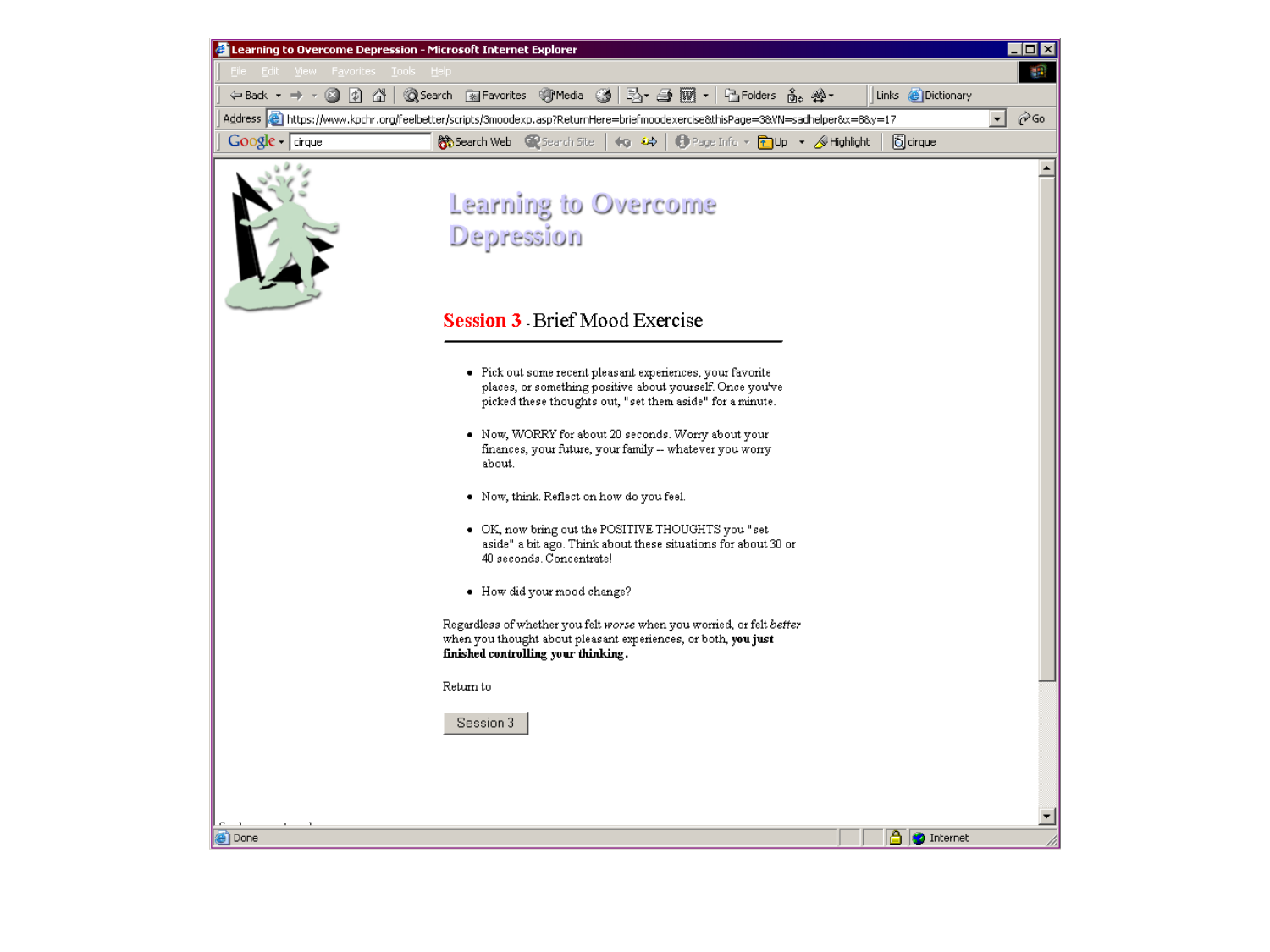

## Slide 8
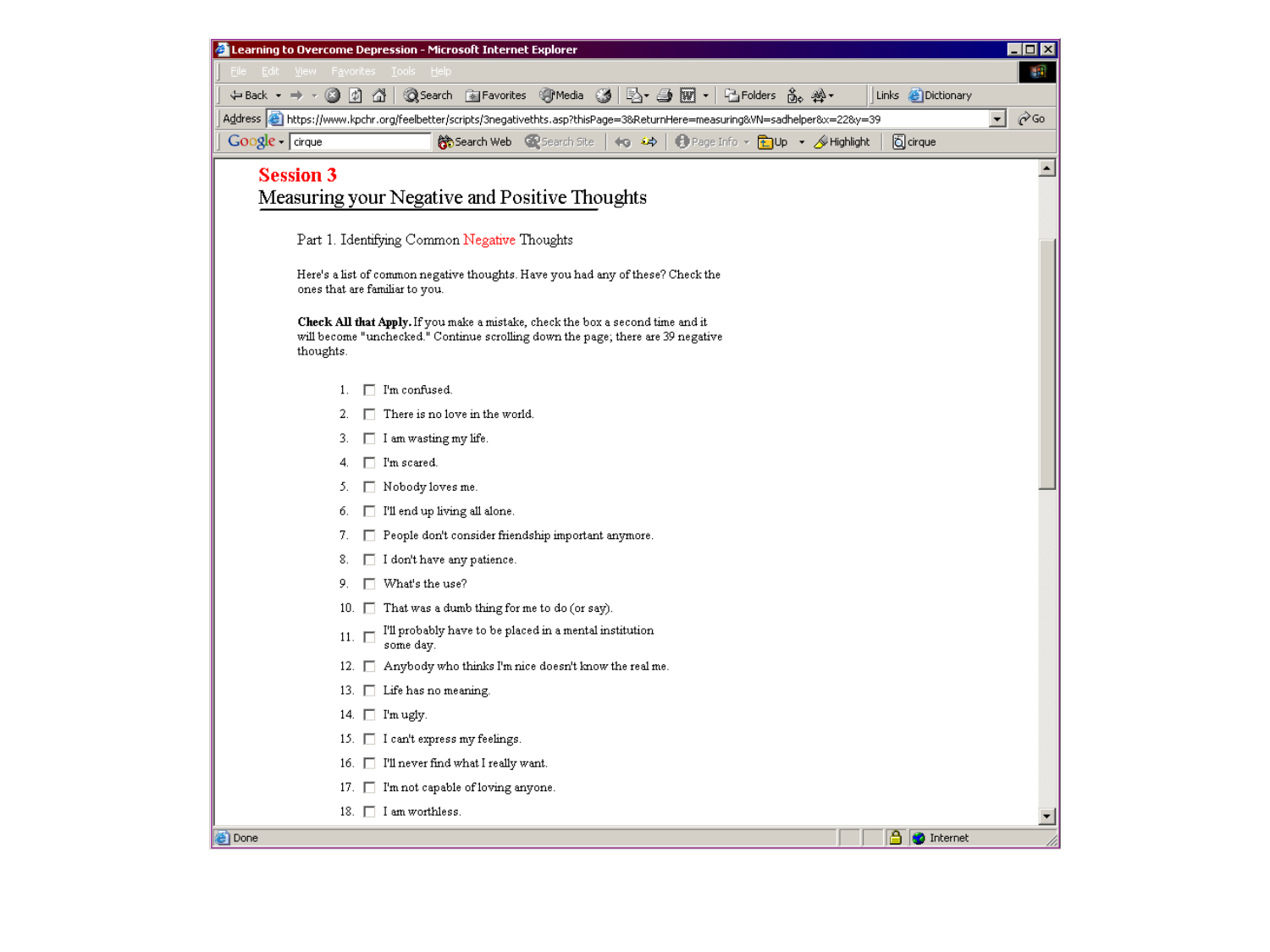

## Slide 9
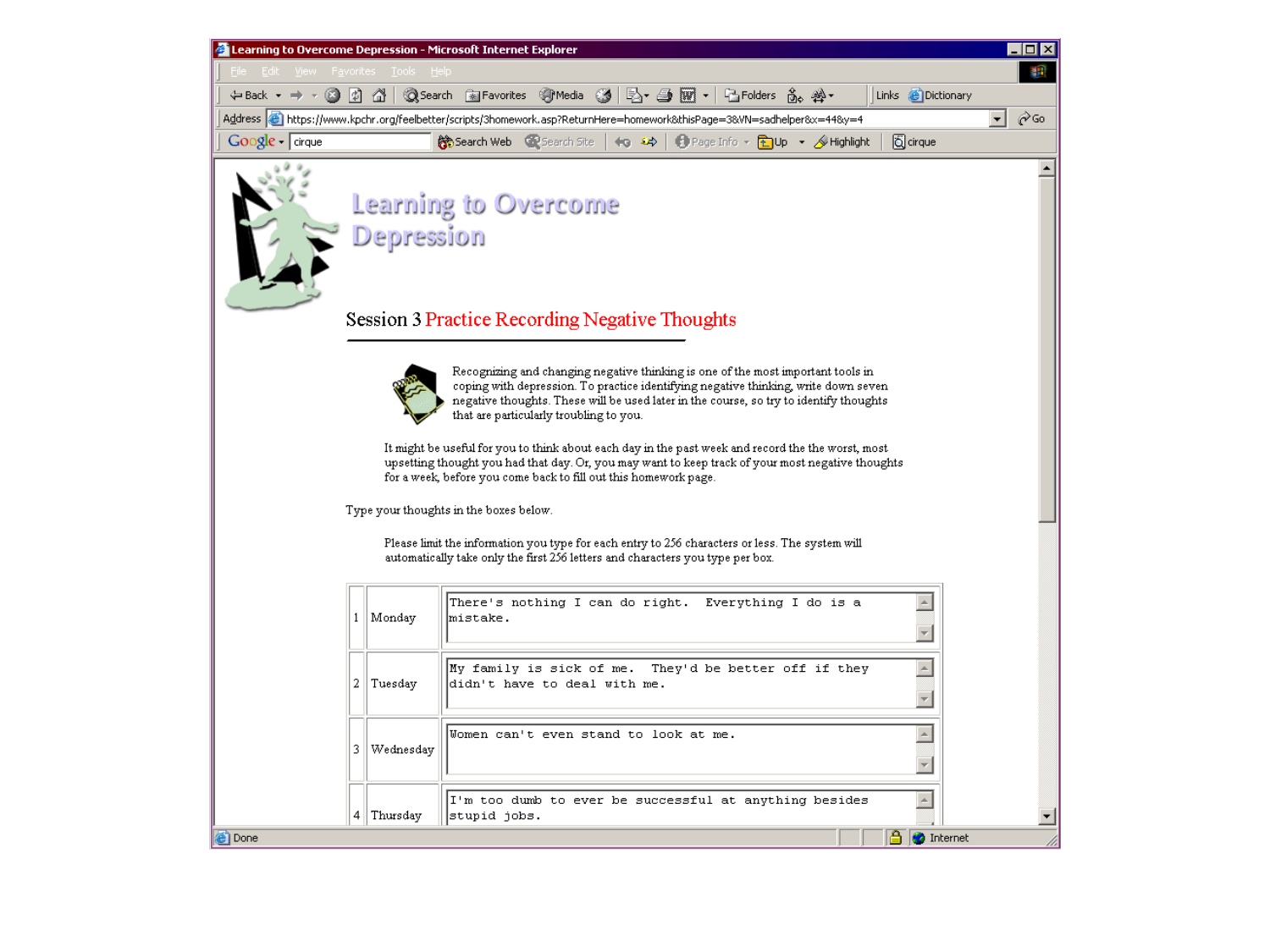

## Slide 10
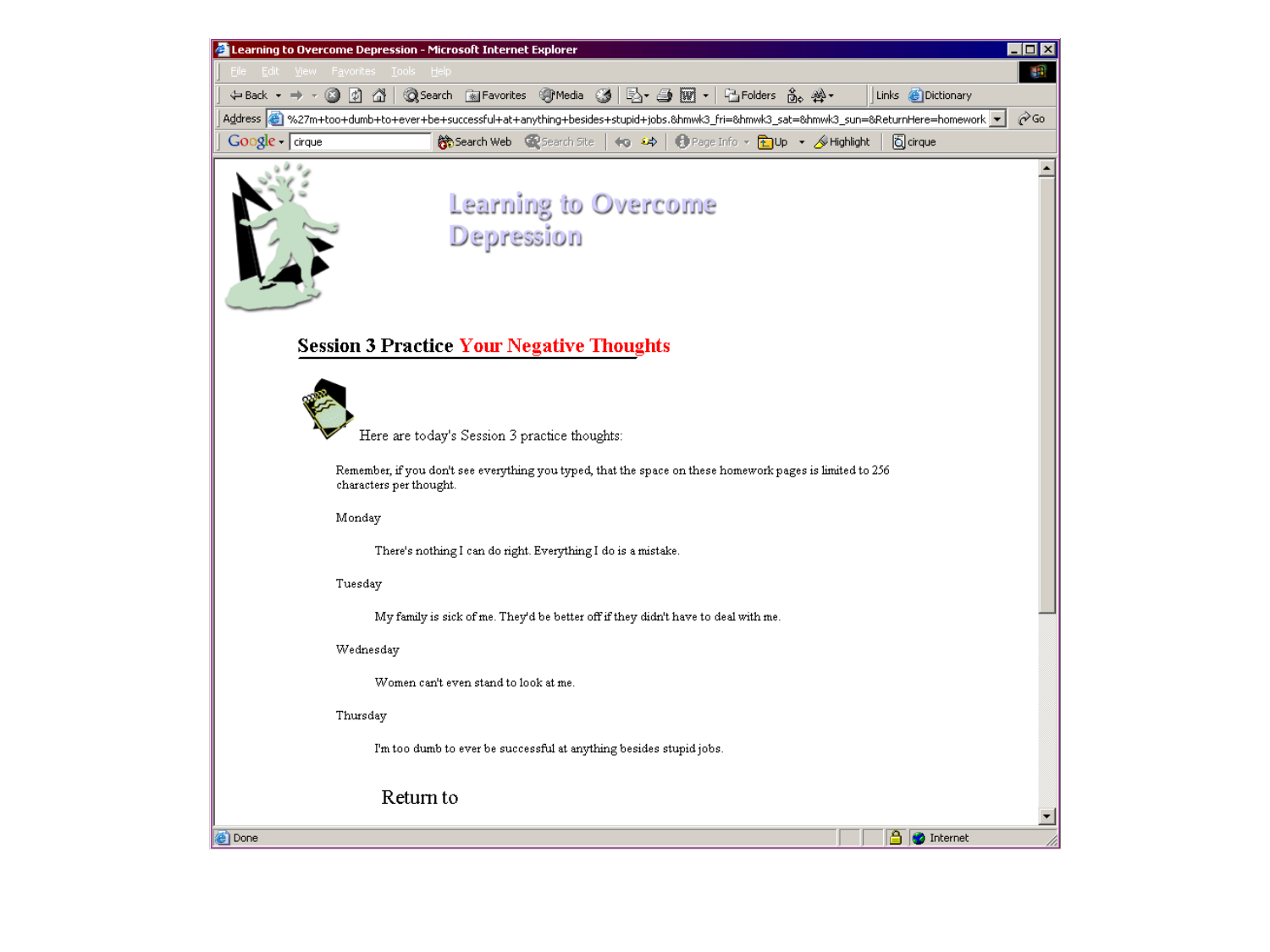

## Slide 11
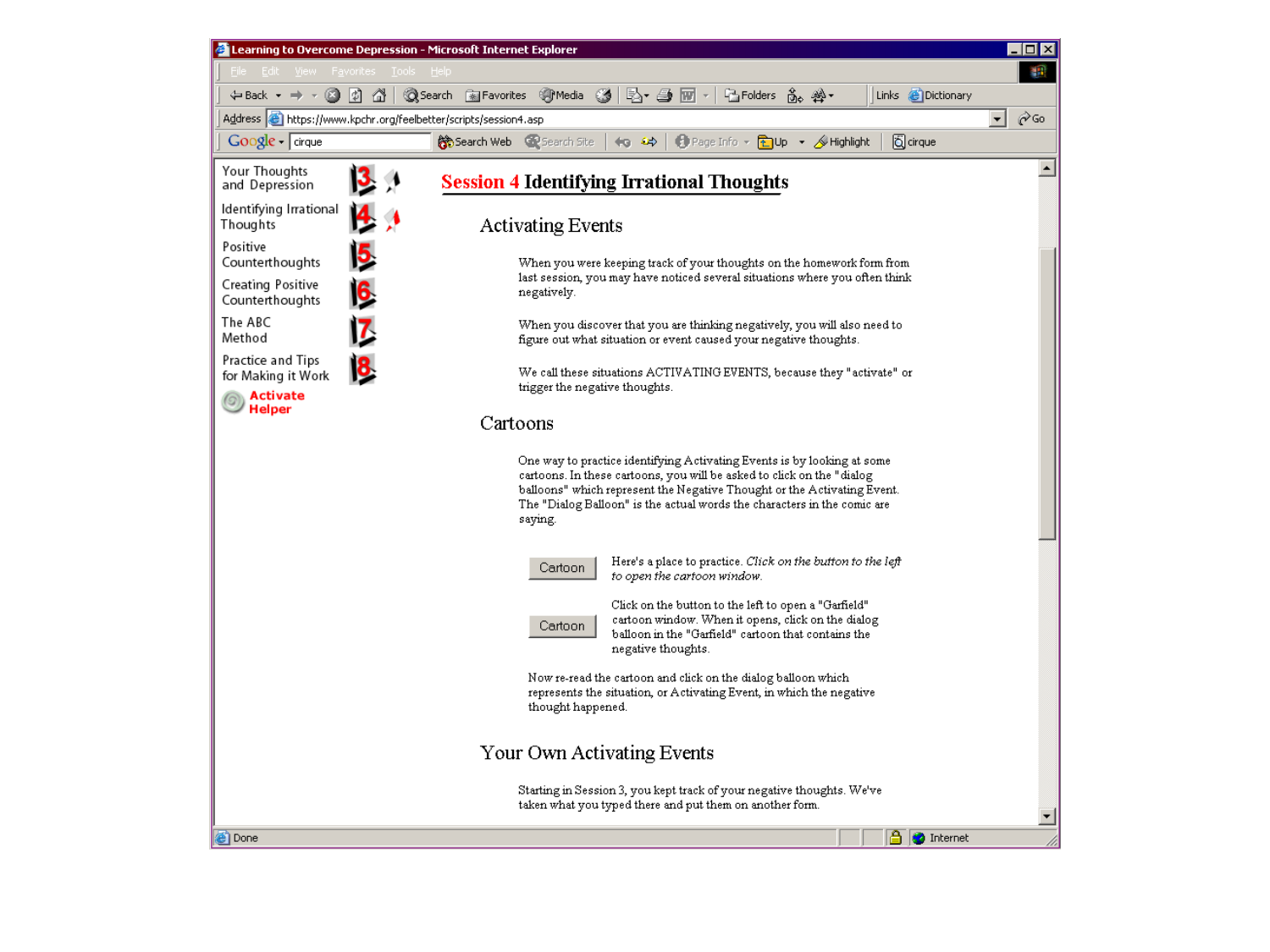

## Slide 12
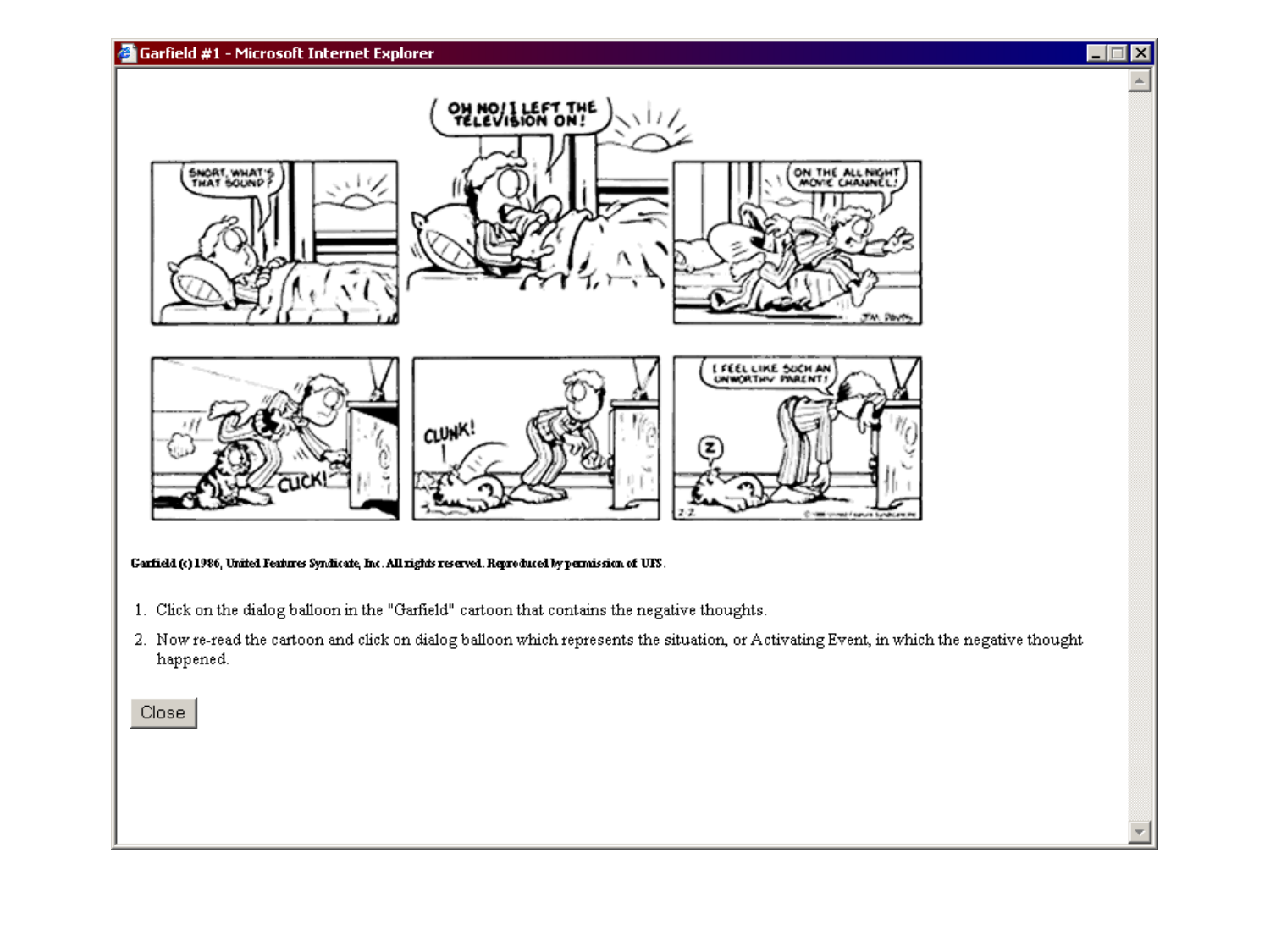

## Slide 13
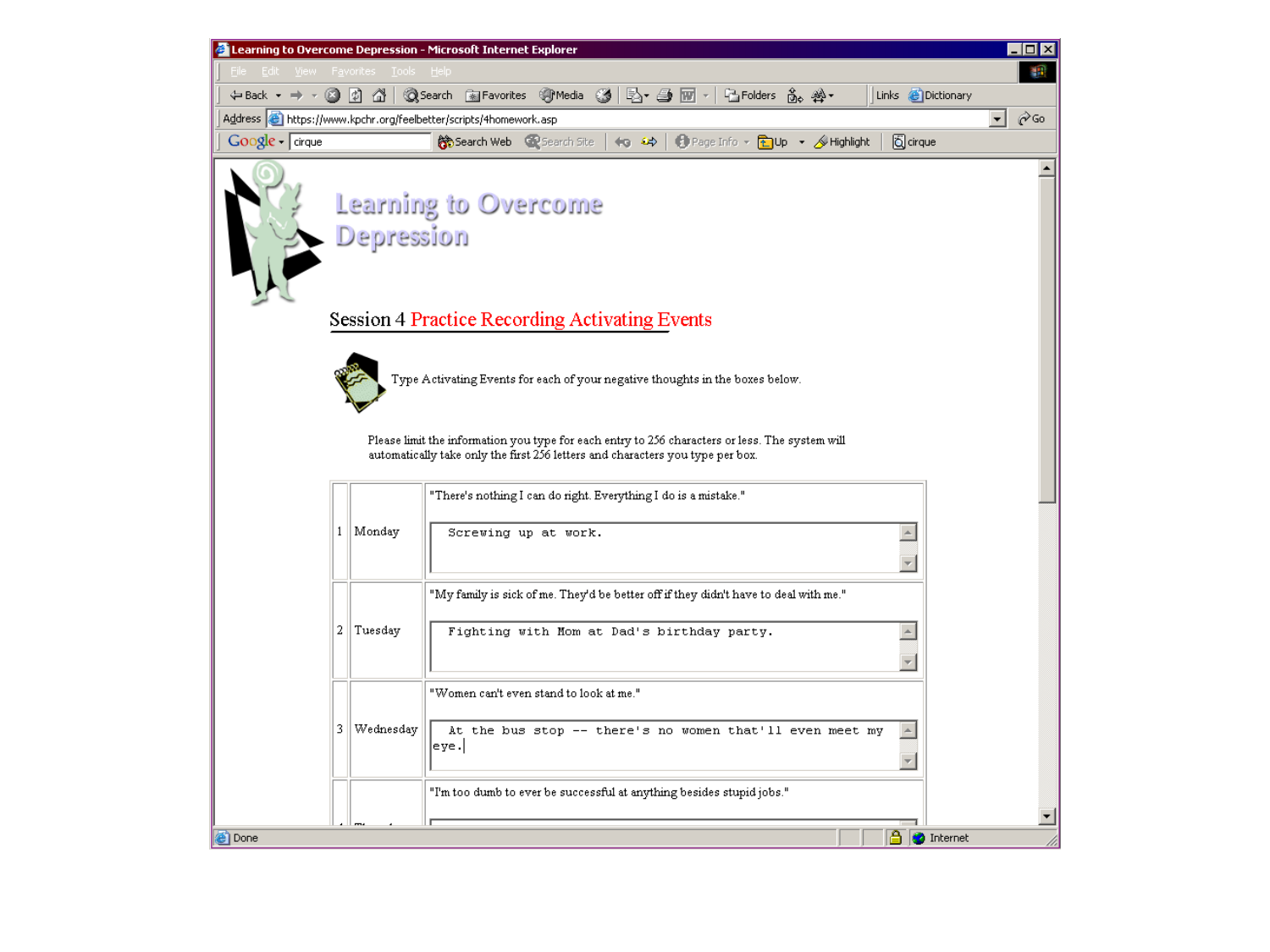

## Slide 14
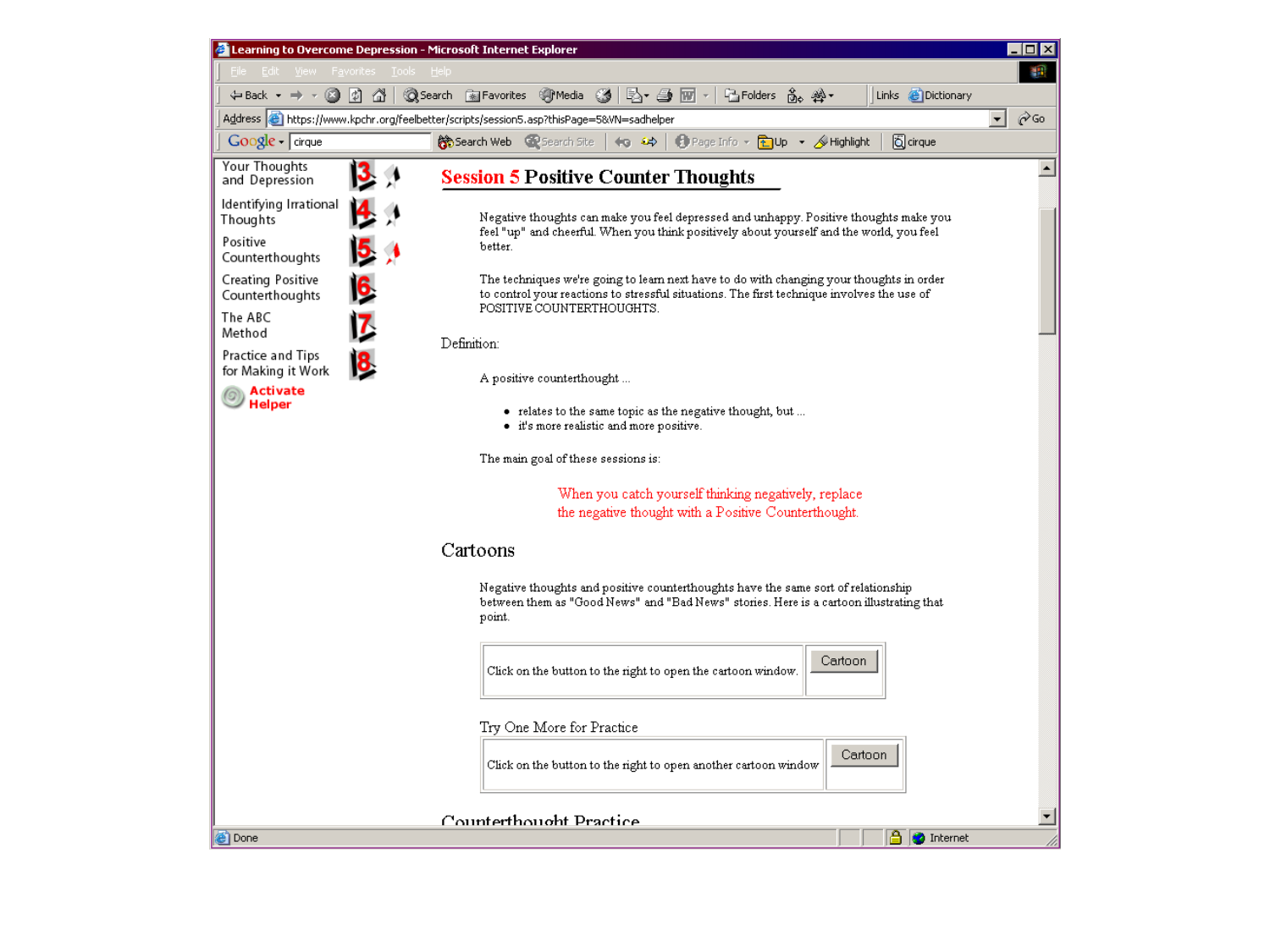

## Slide 15
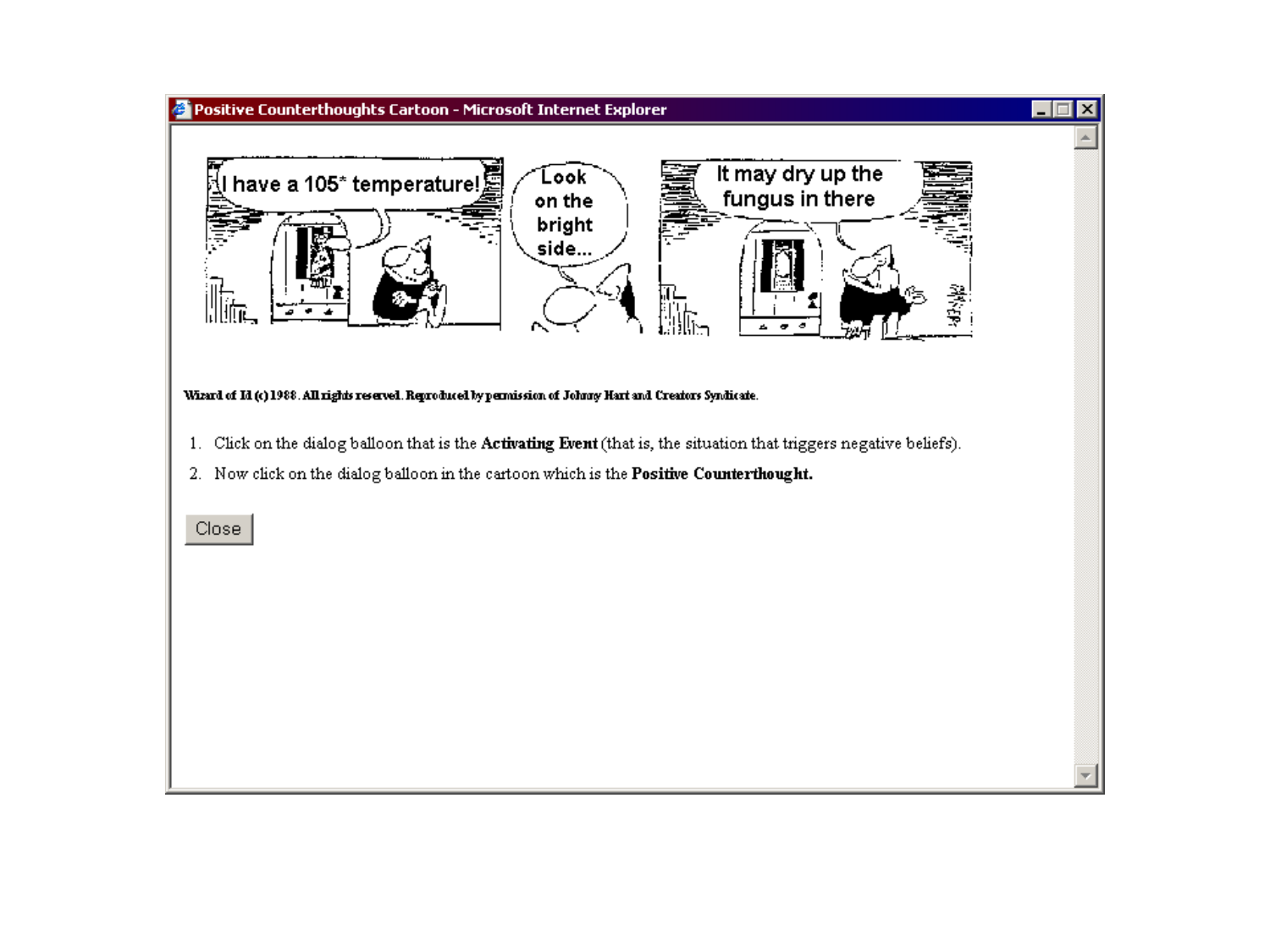

## Slide 16
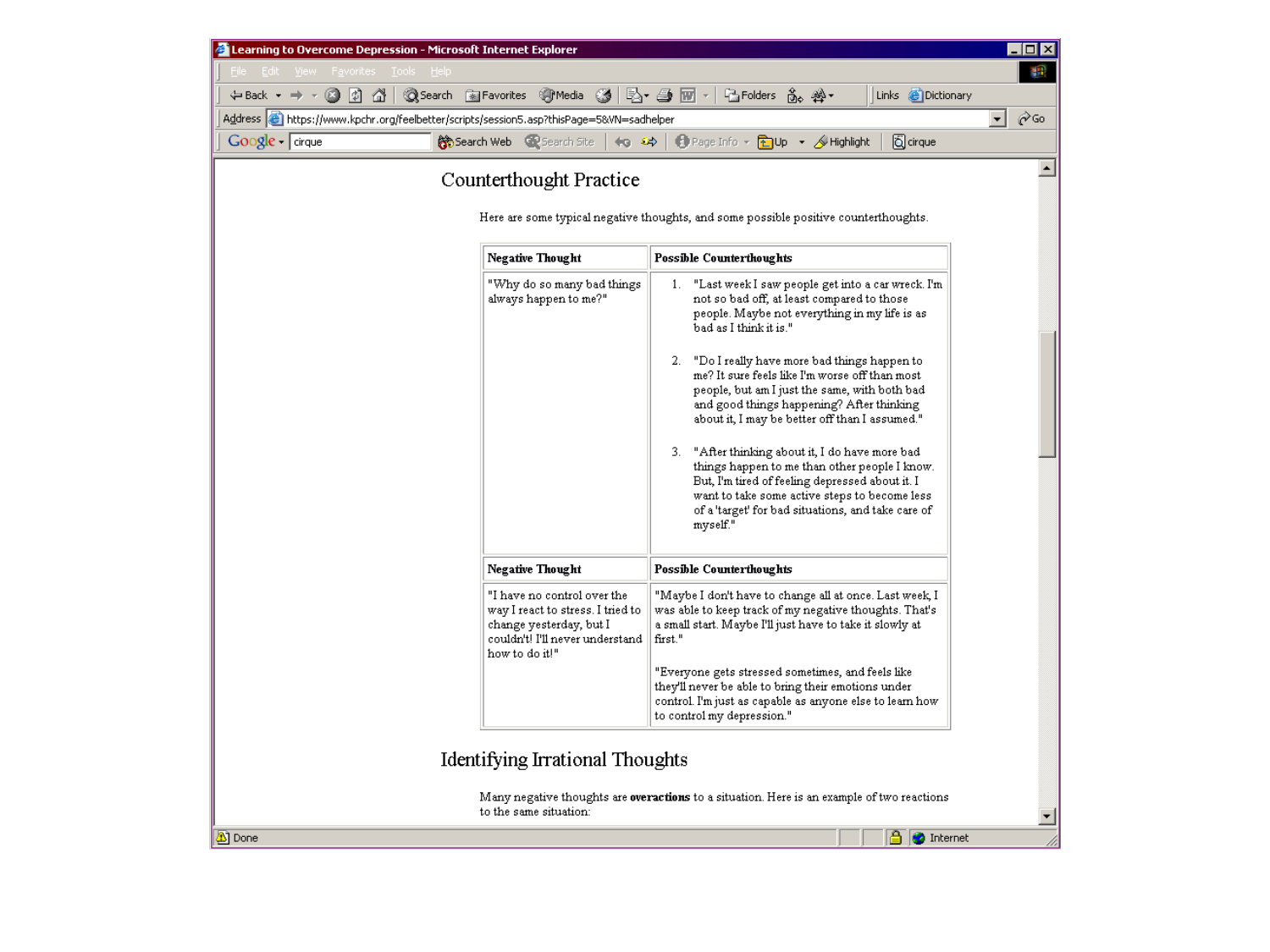

## Slide 17
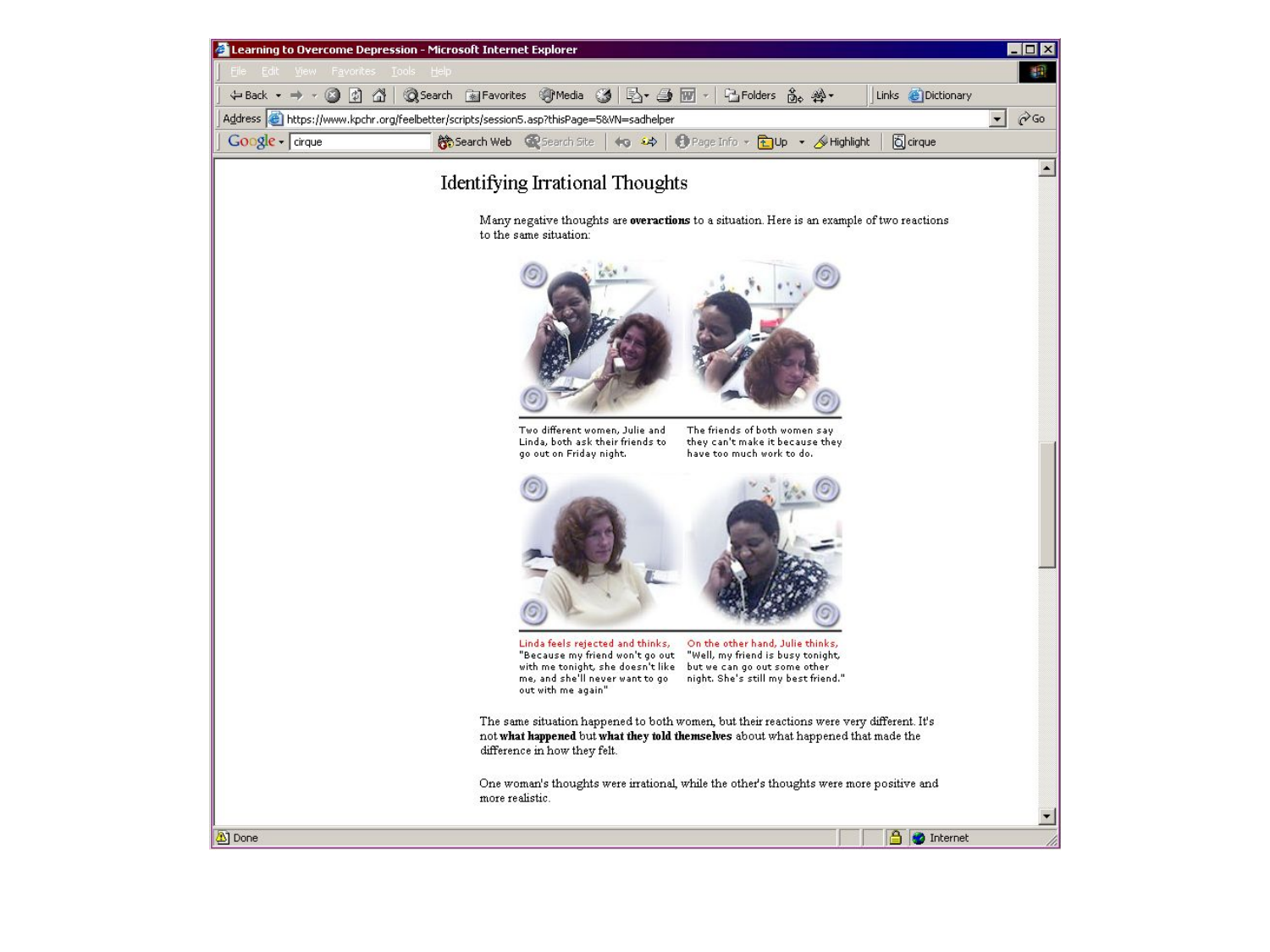

## Slide 18
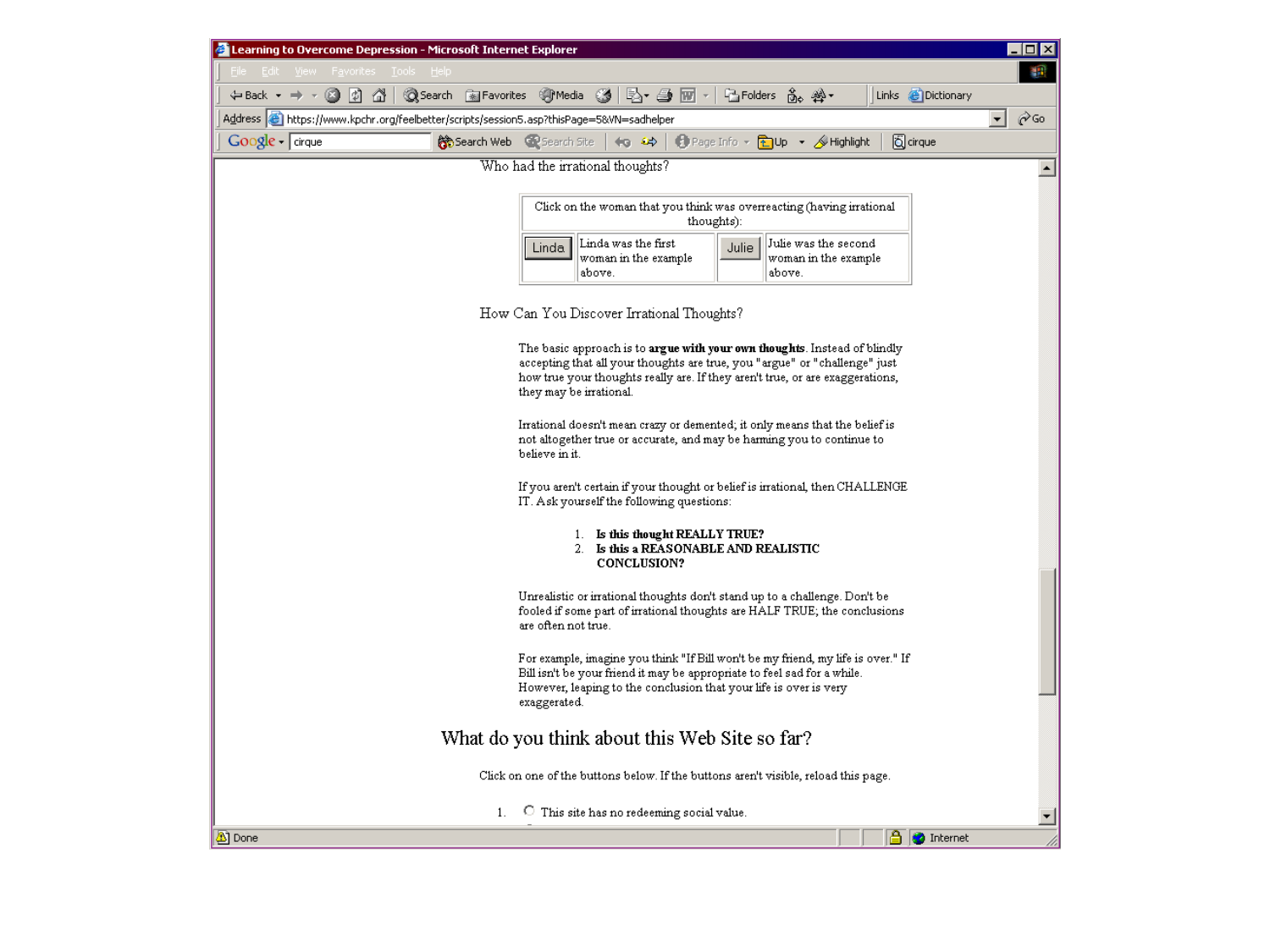

## Slide 19
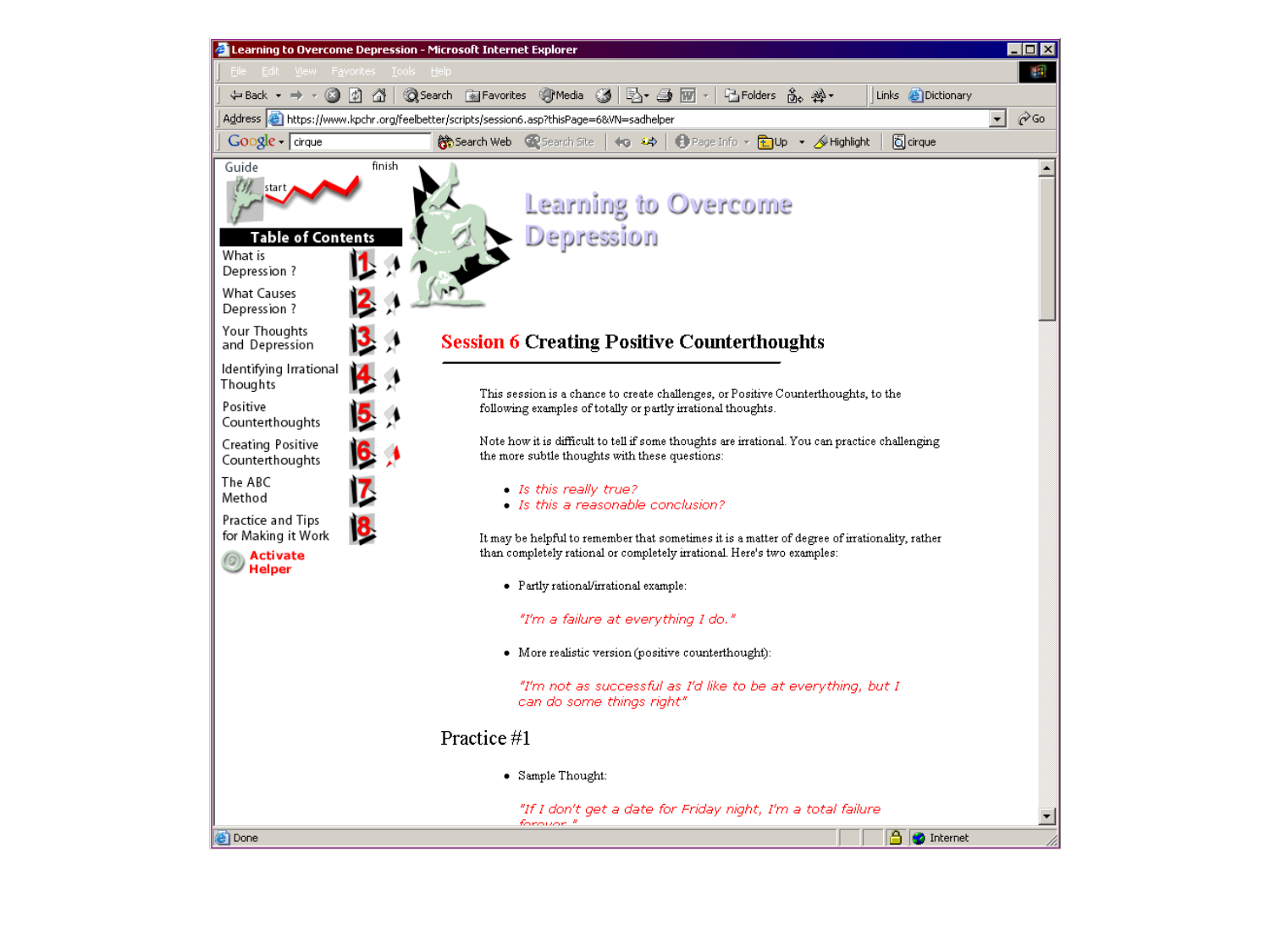

## Slide 20
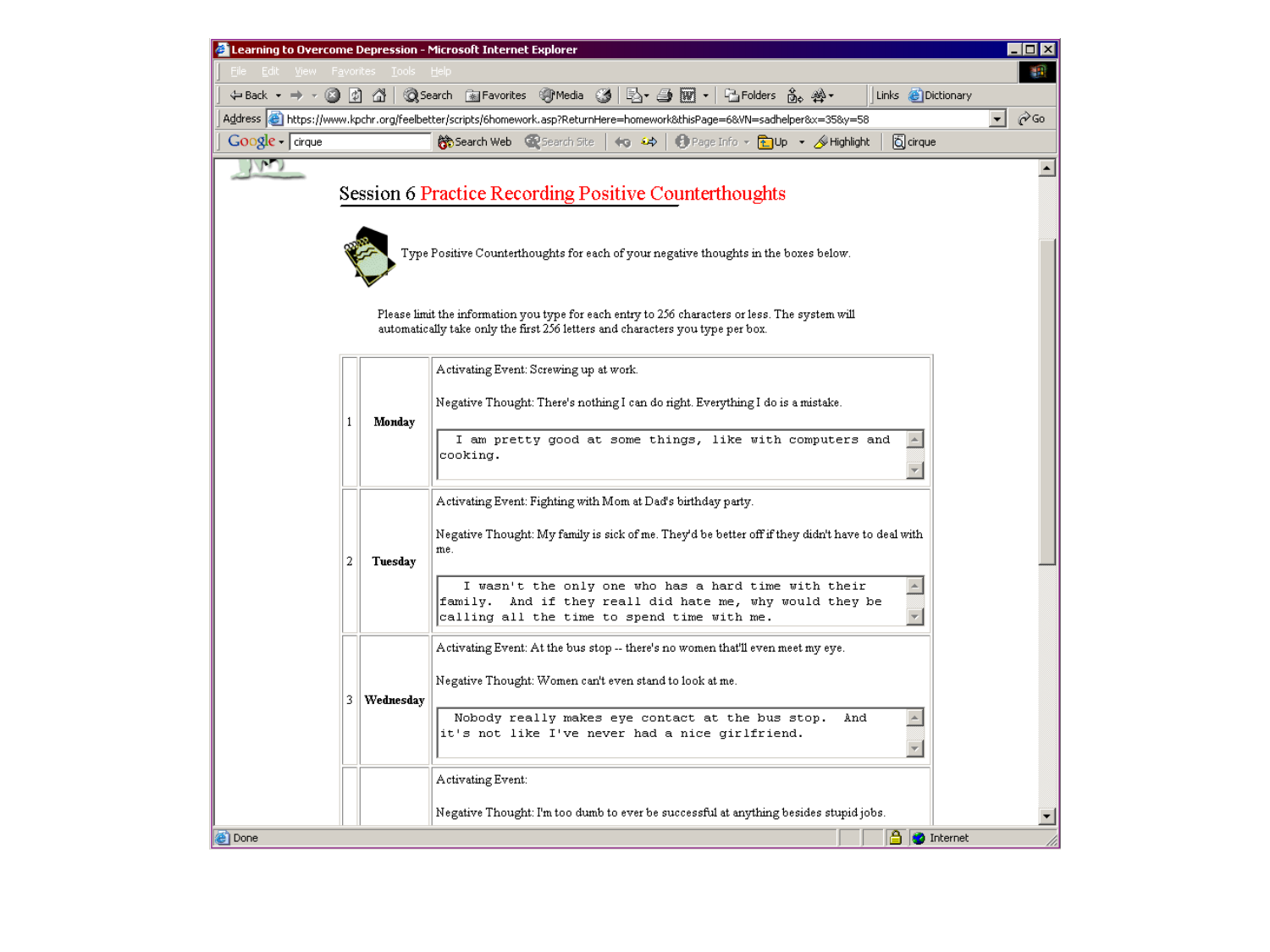

## Slide 21
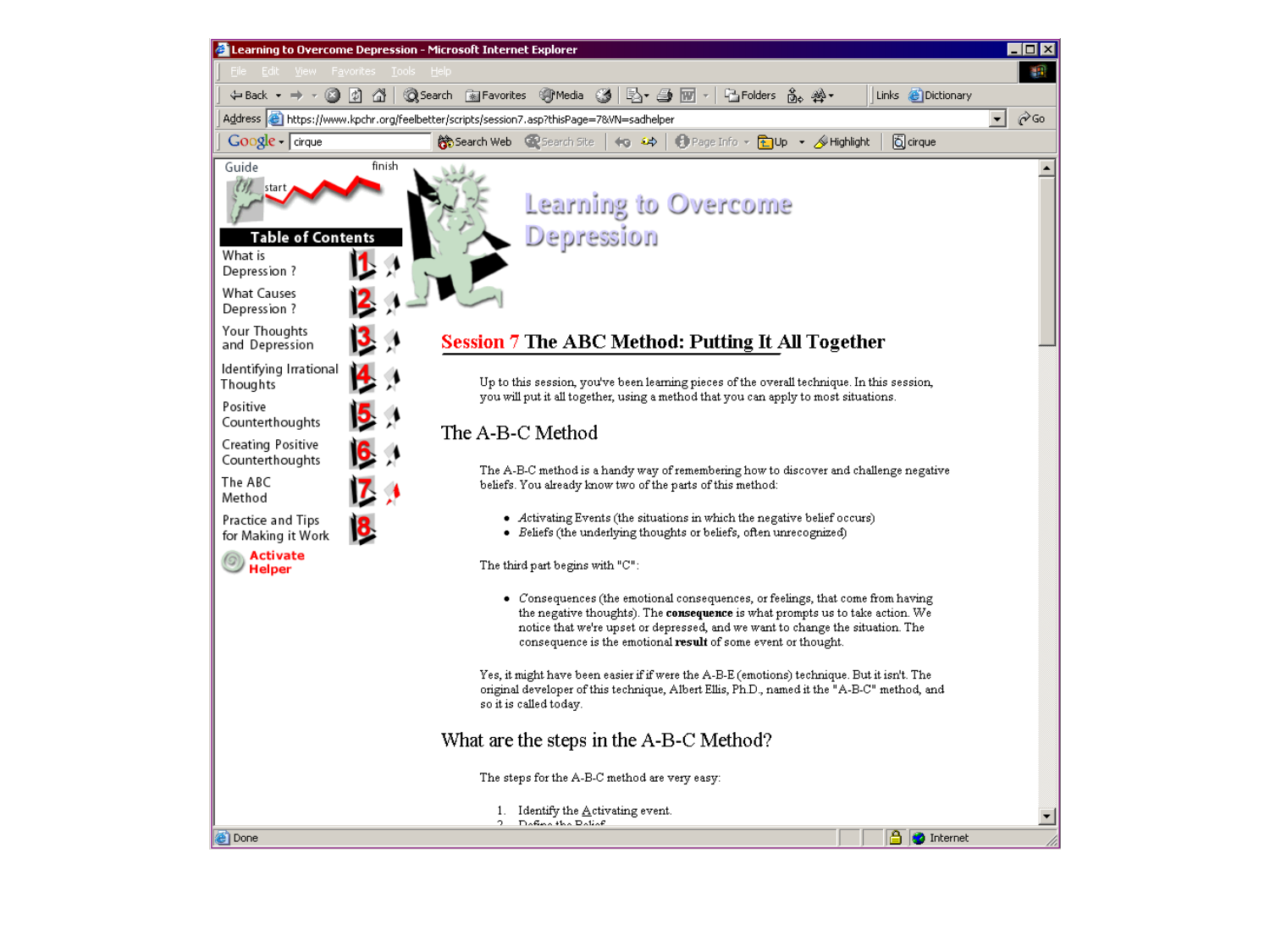

## Slide 22
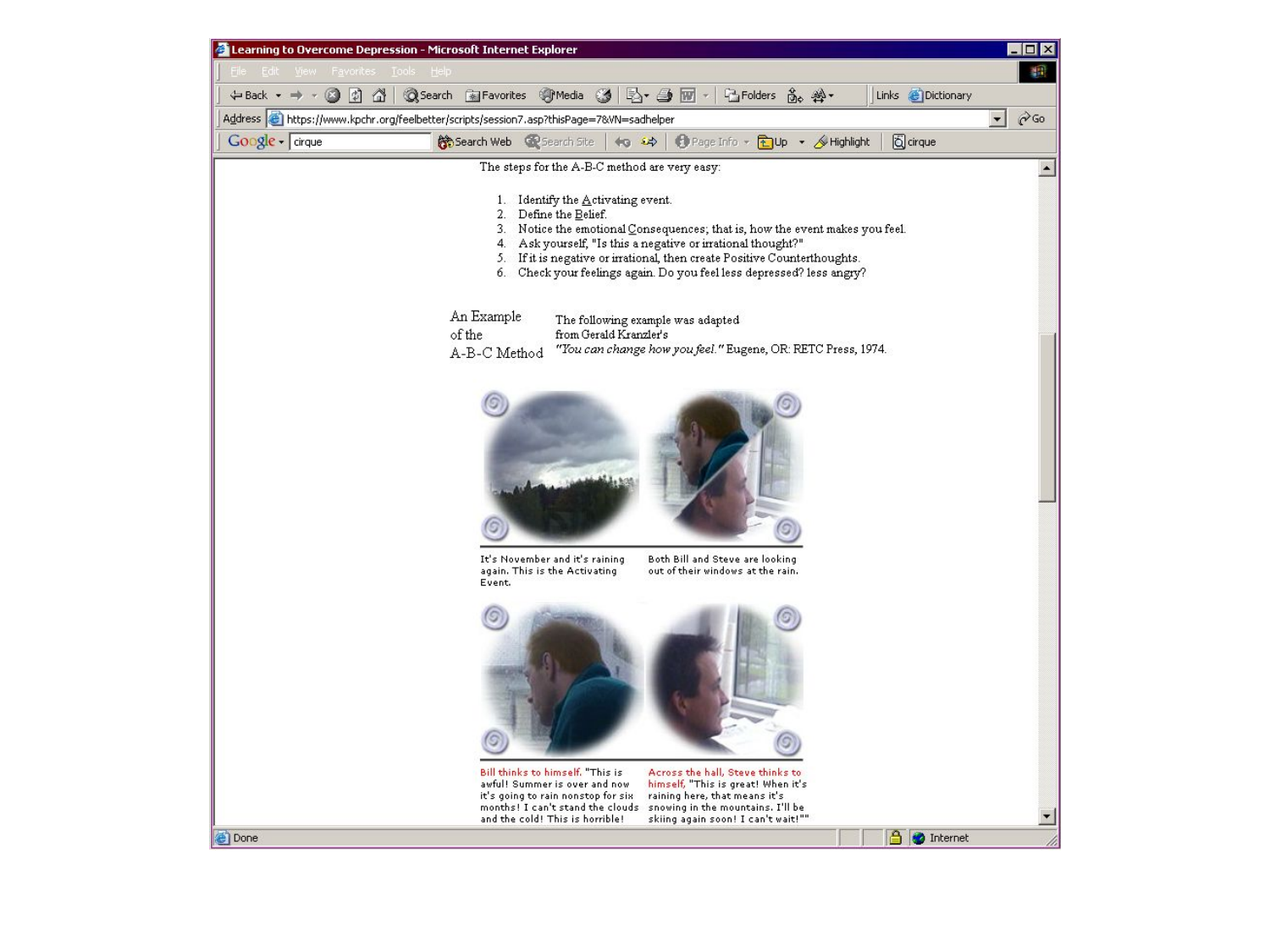

## Slide 23
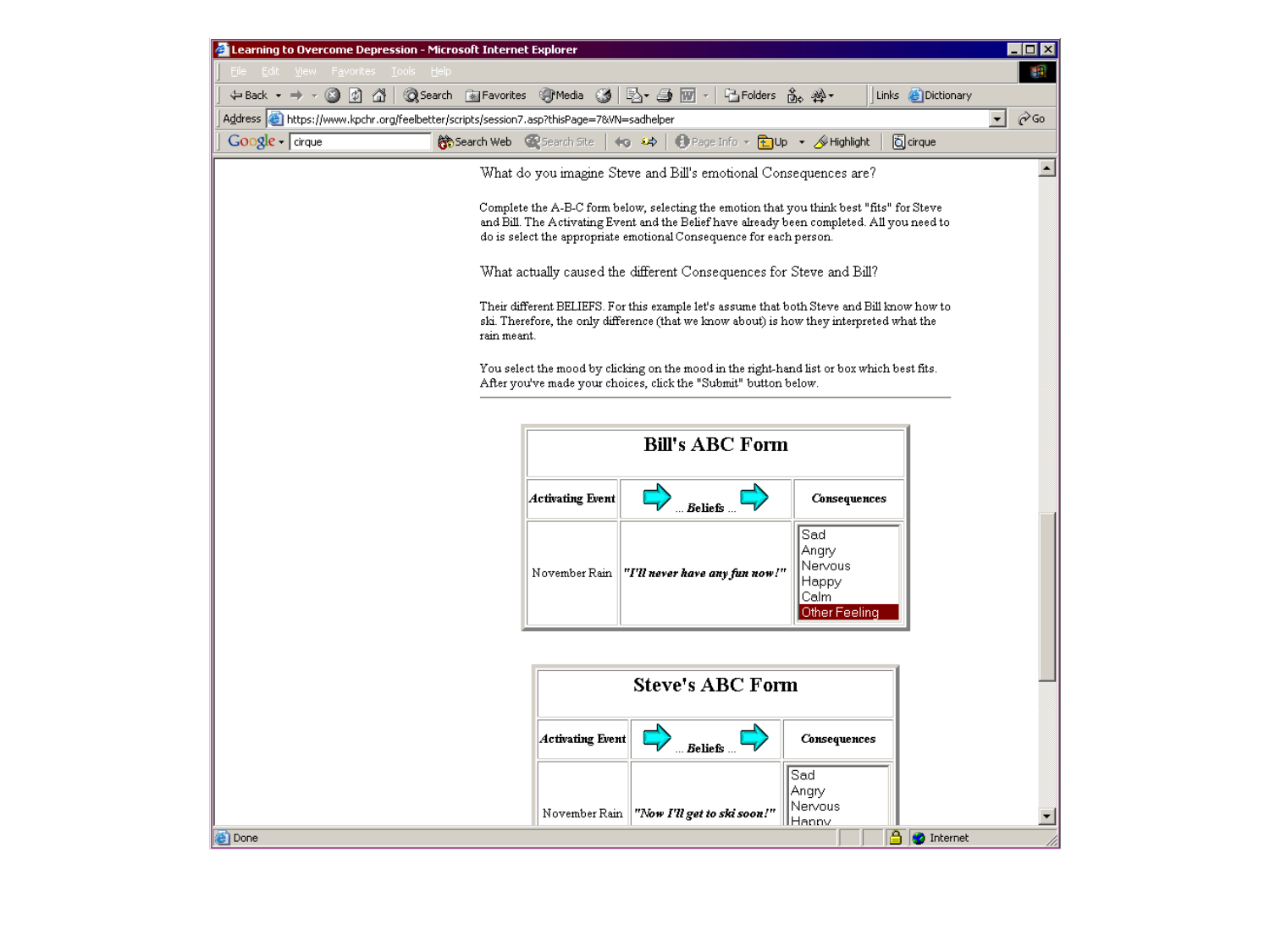

## Slide 24
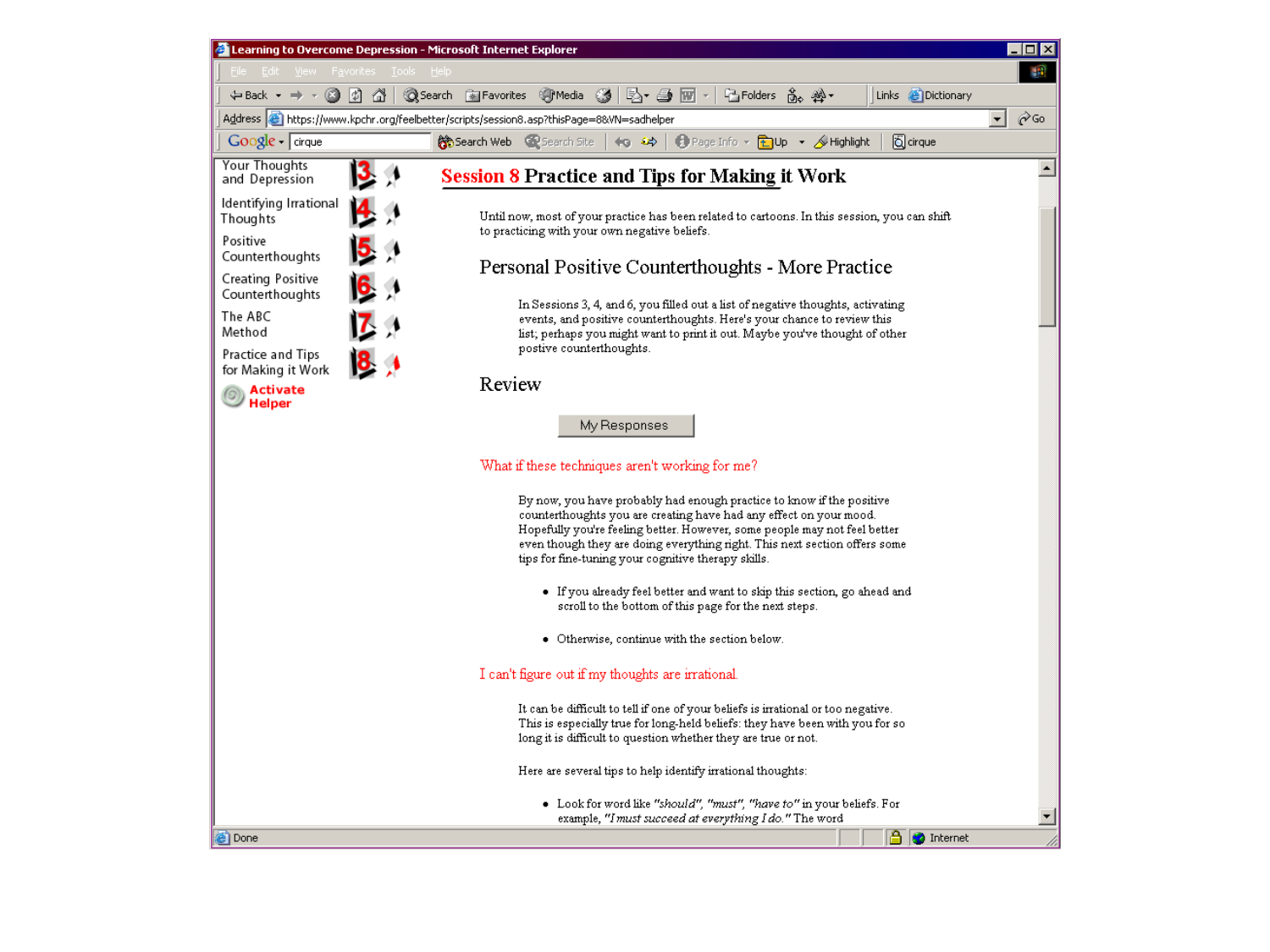

## Slide 25
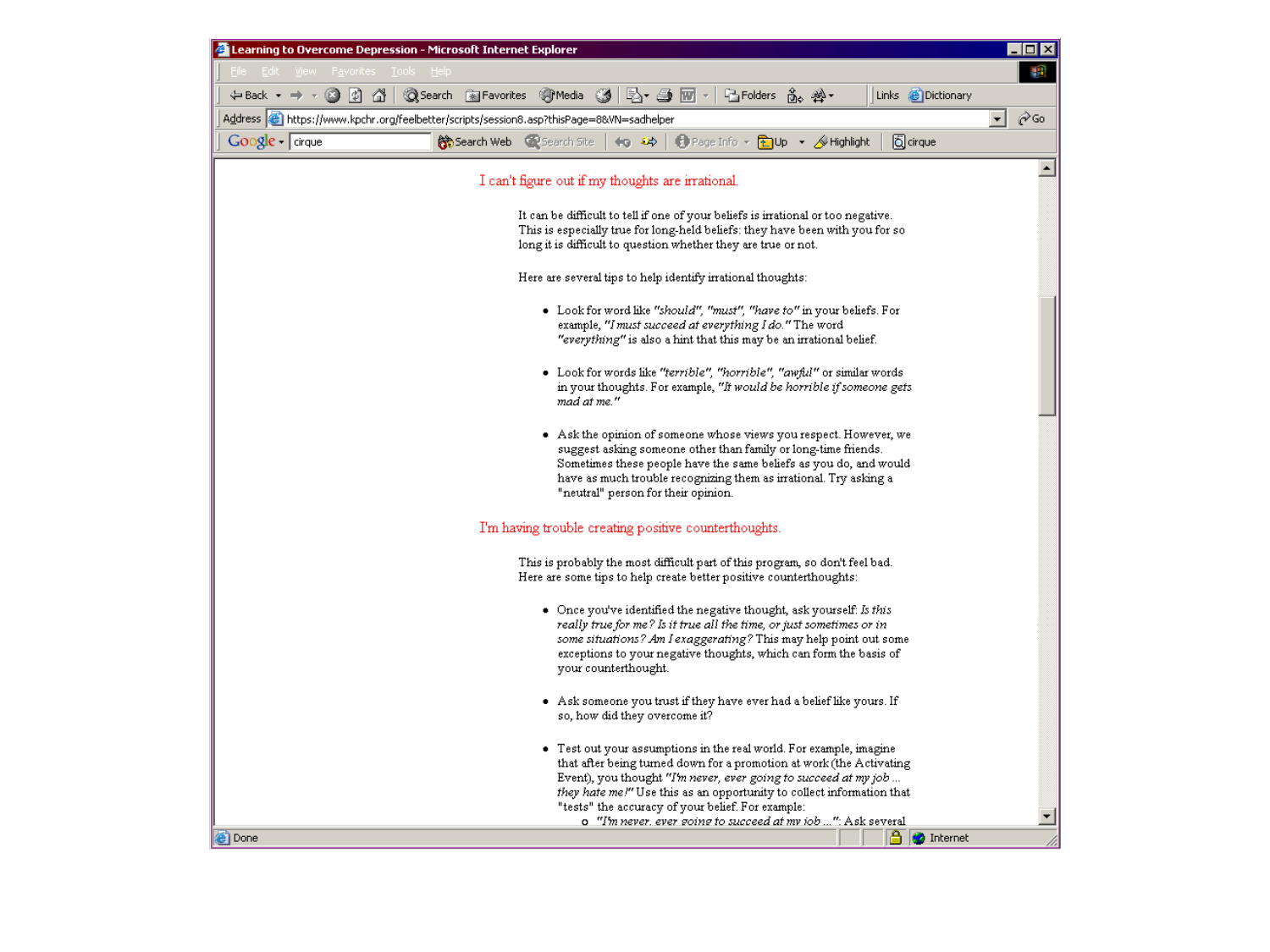

## Slide 26
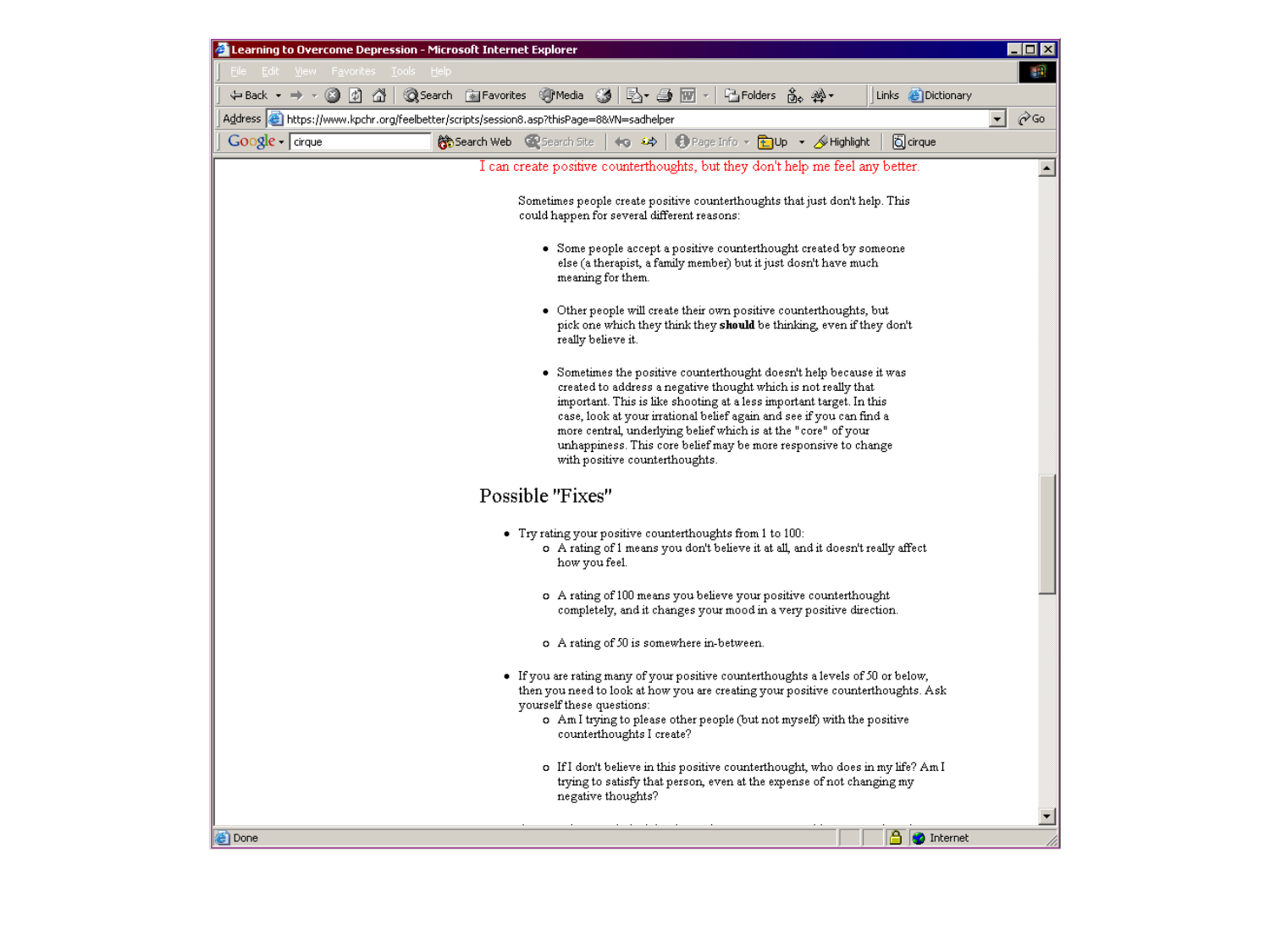

## Slide 27
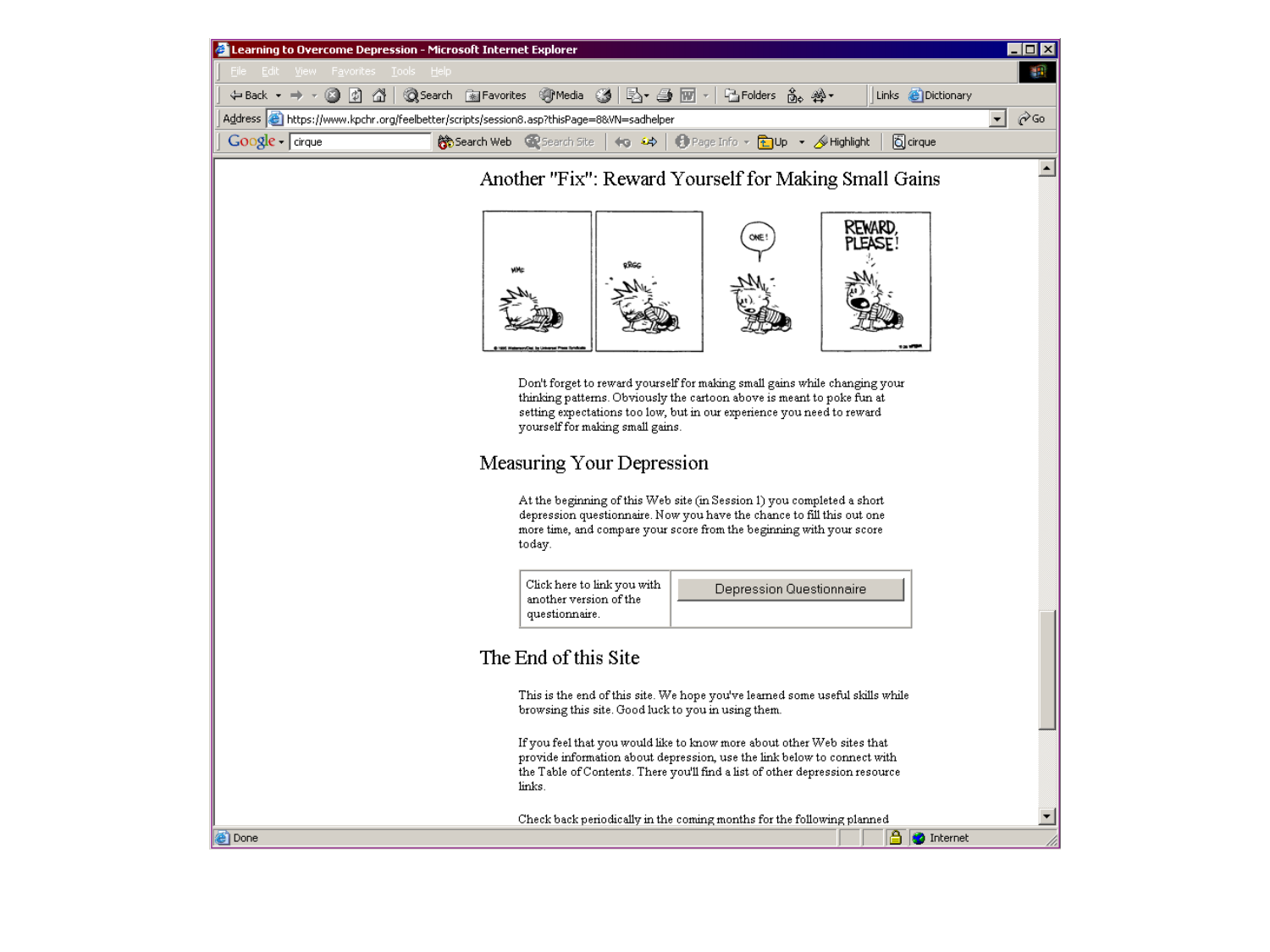

## Slide 28
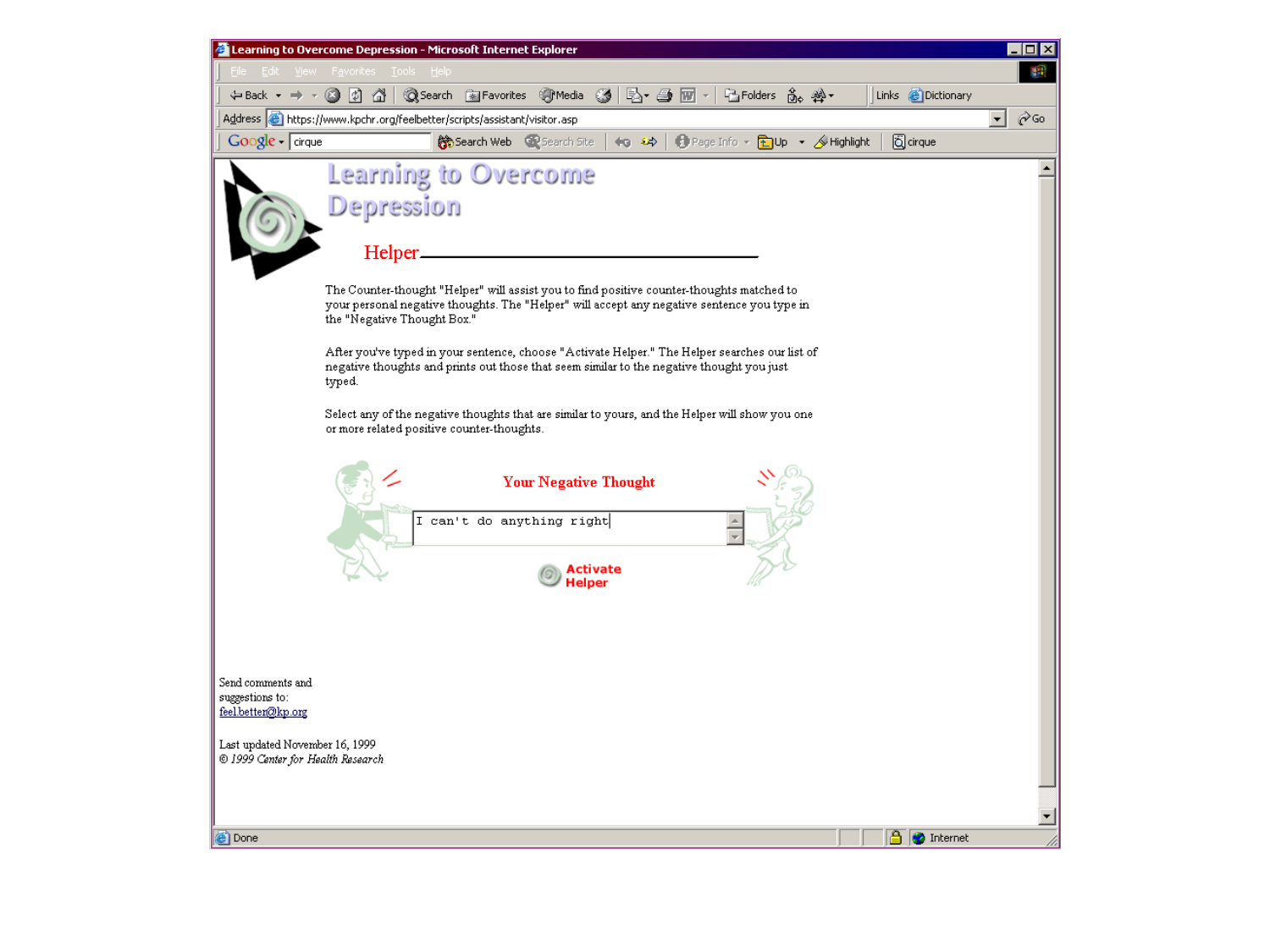

## Slide 29
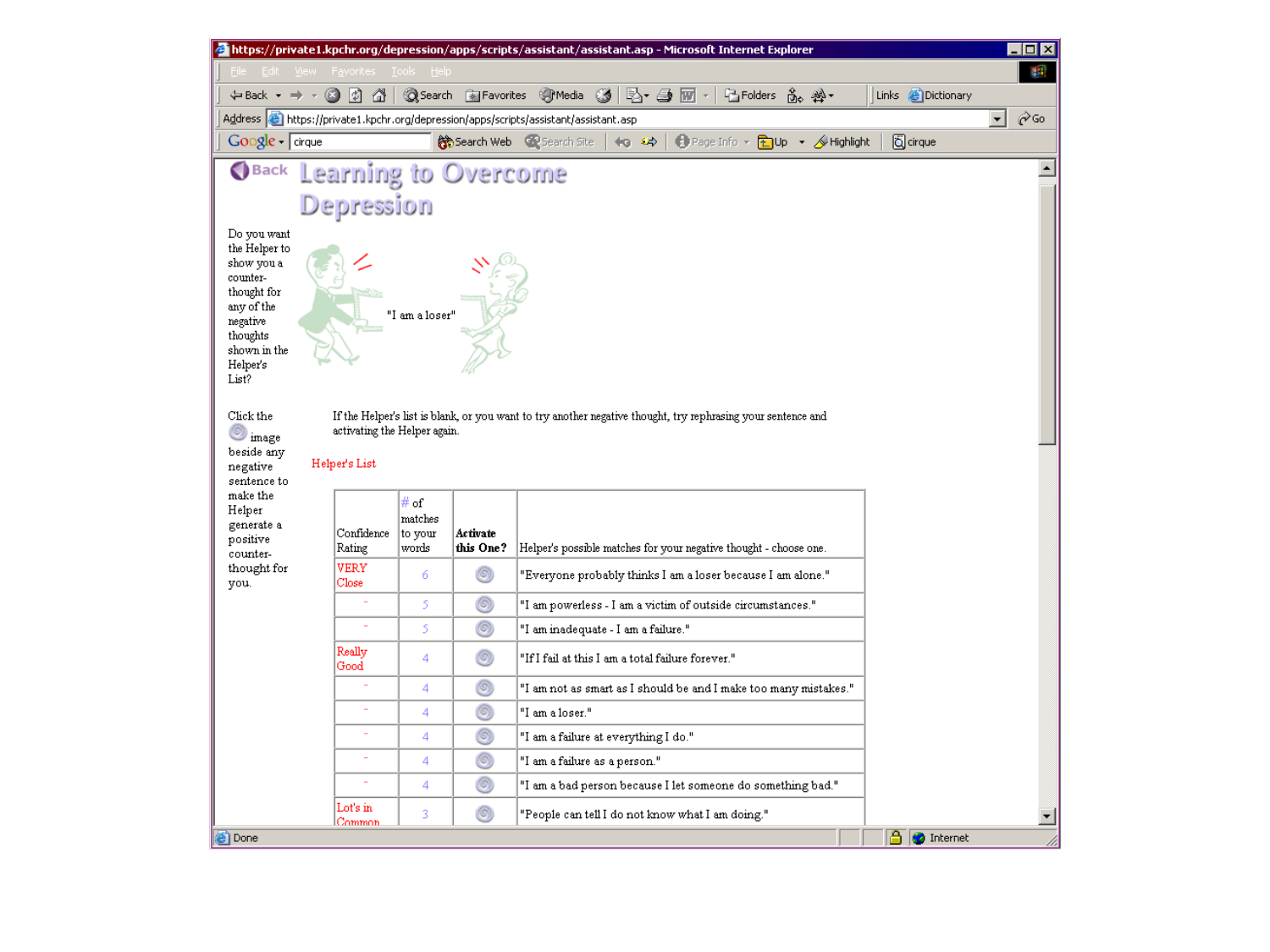

## Slide 30
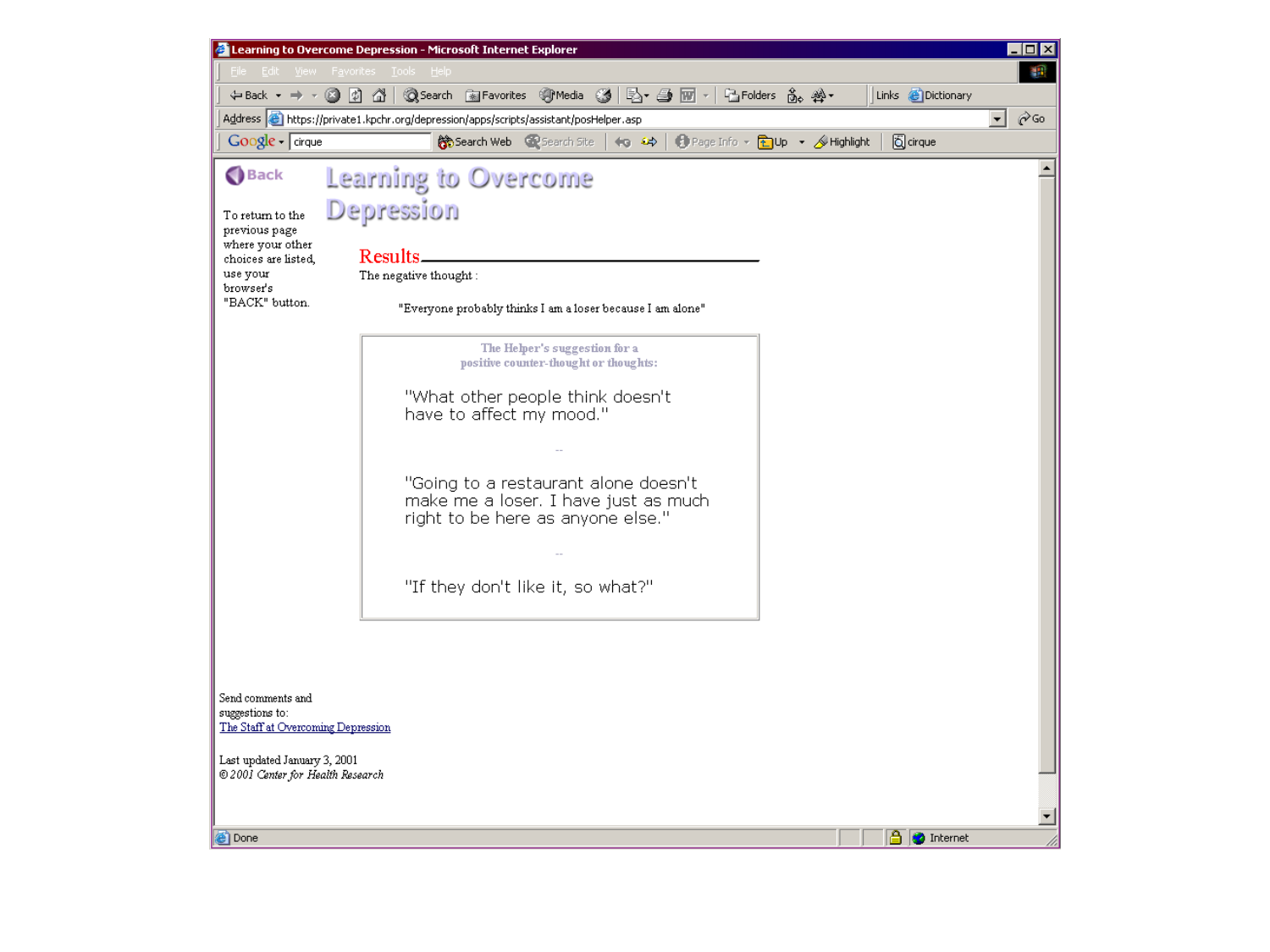

Supplement: Supplementary file 1 [file jmir_v4i3e14_app1.ppt]
